# Molecular Chaperone Calnexin Regulates the Function of *Drosophila* Sodium Channel Paralytic

**DOI:** 10.3389/fnmol.2017.00057

**Published:** 2017-03-07

**Authors:** Xi Xiao, Changyan Chen, Tian-Ming Yu, Jiayao Ou, Menglong Rui, Yuanfen Zhai, Yijing He, Lei Xue, Margaret S. Ho

**Affiliations:** ^1^Research Center for Translational Medicine, Shanghai East Hospital, Tongji University School of MedicineShanghai, China; ^2^Key Laboratory of Arrhythmias of the Ministry of Education of China, Shanghai East Hospital, Tongji University School of MedicineShanghai, China; ^3^Department of Anatomy and Neurobiology, Tongji University School of MedicineShanghai, China; ^4^Shanghai Key Laboratory of Signaling and Diseases Research, School of Life Science and Technology, Institute of Intervention Vessel, Shanghai 10th People's Hospital, Tongji UniversityShanghai, China; ^5^Key Laboratory of Developmental Genes and Human Disease, Institute of Life Sciences, Southeast UniversityNanjing, China

**Keywords:** Calnexin chaperone, locomotor behavior, NMJ synaptogenesis, paralytic, sodium channel

## Abstract

Neuronal activity mediated by voltage-gated channels provides the basis for higher-order behavioral tasks that orchestrate life. Chaperone-mediated regulation, one of the major means to control protein quality and function, is an essential route for controlling channel activity. Here we present evidence that *Drosophila* ER chaperone Calnexin colocalizes and interacts with the α subunit of sodium channel Paralytic. Co-immunoprecipitation analysis indicates that Calnexin interacts with Paralytic protein variants that contain glycosylation sites Asn313, 325, 343, 1463, and 1482. Downregulation of Calnexin expression results in a decrease in Paralytic protein levels, whereas overexpression of the Calnexin C-terminal calcium-binding domain triggers an increase reversely. Genetic analysis using adult climbing, seizure-induced paralysis, and neuromuscular junction indicates that lack of Calnexin expression enhances Paralytic-mediated locomotor deficits, suppresses Paralytic-mediated ghost bouton formation, and regulates minature excitatory junction potentials (mEJP) frequency and latency time. Taken together, our findings demonstrate a need for chaperone-mediated regulation on channel activity during locomotor control, providing the molecular basis for channlopathies such as epilepsy.

## Introduction

Neurological channelopathies such as epilepsy or hyperekplexia pertaining ion channel dysfunctions in the nervous system are characterized with irregular neuronal excitability and behavioral anomalies (Kullmann and Waxman, 2010). Recent studies have implicated the endoplasmic reticulum-associated degradation (ERAD) as one of the regulatory mechanisms for channelopathies (Hoseki et al., 2010). Major ERAD effectors, the ER lectin-like chaperones Calnexin (Cnx), have been shown to exhibit important roles in regulating the folding and stability of channel proteins (Buck et al., 2010; Faria et al., 2012; Young, 2014). Cnx, together with its soluble paralog Careticulin (CRT) and the thiol oxidoreductase ERp57 that catalyzes disulfide bond formation, comprise the ER Cnx/CRT cycle that mediates glycoprotein folding (Williams, 2006; Caramelo and Parodi, 2008; Lederkremer, 2009). The ER-luminal portion of Cnx contains a globular lectin domain which mediates the interaction with the oligosaccharide intermediate Glc_1_Man_9_GlcNAc_2_ on the glycoprotein, and an arm domain that associates with ERp57 at the tip. Cnx also possesses binding sites for ATP, Ca^2+^, and non-native polypeptides (Williams, 2006; Brockmeier et al., 2009; Zhang et al., 2009). These interactions add layers of complexity to the regulatory and binding modes with substrate glycoproteins. Intriguingly, in addition to a long list of substrates such as the immunological surface molecule Class I major histocompatibility complex (MHC) (Margolese et al., 1993; Jackson et al., 1994; Vassilakos et al., 1996), channel proteins such as the epithelial sodium channel (ENaC), the potassium channel Shaker, and the cystic fibrosis transmembrane conductance regulator (CFTR), have also been suggested as potential Cnx substrates (Higgins et al., 2003; Okiyoneda et al., 2004; Buck et al., 2010). These studies raise the possibility that Cnx is one of the major regulators for ion channel function and serves as a therapeutic target for treating symptoms associated with channelopathies such as behavioral anomalies.

The animal model *Drosophila melanogaster* has been frequently used as a disease paradigm for its rapid and easy access to genetic tools and behavioral analysis. Based on previous studies and sequence blasts, three *Drosophila cnx* homologs: *cnx11A, cnx14D*, and *cnx99A*, have been predicted and reported (Hong and Ganetzky, 1996; Christodoulou et al., 1997; McGovern et al., 2003). Among these three genes, *cnx99A* (henceforth *cnx*) exhibits the highest similarity with the mammalian *cnx* (Rosenbaum et al., 2006), hence the focus of the current study. Similar to vertebrates, *Drosophila* Cnx contains a N-terminal ER luminal domain, a transmembrane (TM) domain, and a cytosolic domain. The Cnx ER luminal domain contains two distinct regions: a compact, globular lectin domain and a proline-rich P domain, whereas the cytosolic domain contains a sole calcium-binding domain (Schrag et al., 2001; Rosenbaum et al., 2006). Interestingly, *Drosophila* Cnx regulates rhodopsin maturation and calcium buffering in compound eyes, demonstrating a role as both a molecular chaperone and Ca^2+^ regulator (Rosenbaum et al., 2006; Tian et al., 2013). Here we show that *Drosophila* Cnx colocalizes and interacts with the α subunit of sodium channel Paralytic (Para) in adult brains. Cnx-Para interaction depends on Para glycosylation sites. Lack of Cnx expression leads to a decrease in Para protein levels, whereas overexpression of its C-terminal calcium-binding domain triggers an increase. Cnx-mediated Para function is required for locomotor behavior such as adult climbing and seizure-induced paralysis, and underlies synaptic differentiation. These findings advance our understanding for diseases associated with dysfunction of channel proteins such as epilepsy.

## Materials and methods

### Fly strains

Flies were maintained at 25°C on normal food. All strains were obtained from Bloomington Stock Center, VDRC, or as gifts from colleagues. All fly crosses were carried out at 25°C with standard laboratory conditions unless noted otherwise. Transgenic *cnx99A, cnx-N*, and *cnx-C* flies were created by embryonic microinjection performed in the *Drosophila* Technique Platform of Institute of Biochemistry and Cell Biology (SIBS, CAS, China).

### Molecular biology and biochemistry

To generate transgenic *cnx99A* flies, full length of *cnx99A* cDNA (SD17909) from *Drosophila* Genomics Resources Center (DGRC) was first cloned using standard protocols. Primer sequences designed for PCR were: 5′-ATGGCGTGGAAAATGGGAGGAAA-3′ and 3′-TTACTCCTTGCGCGCTTGACGCTT-5′. Purified PCR products were then cloned into the *pUAST-3xFlag* Gateway vector (Invitrogen) to make constructs for S2 cell analysis and transgenic fly microinjection. Next, DNA fragments were amplified by PCR to subclone *cnx-N* (1–1,467 bps, N-terminal luminal domain) and *cnx-C* (1,525–1,818 bps, cytosolic domain) from the full length *cnx*. Primers used were: 5′-GCGTGGAAAATGGGAG -3′ and 5′-TTACGAGGGATTGGCCTTGGC-3′ for *cnx-N*, 5′-AGATTTGGTACCGCTAAGAGTCAG-3′ and 5′-TTACTCCTTGCGCGCTTG-3′ for *cnx-C*. Purified PCR products were cloned similarly as above to make constructs for S2 cell analysis and transgenic fly microinjection.

Furthermore, Para protein variants: fragments between Para domain I segment 5 and 6 that contains three predicted glycosylation sites at Asn313, 325, and 343 (Para_IS5−S6_), Para III domain segment 5 and 6 that contains two predicted glycosylation sites at Asn1463 and 1482 (Para_IIIS5−S6_), Para domain II and III that contains no predicted glycosylation sites (Para_IIS6−IIIS1_), and the first N-terminal intracellular domain of Para (Para-N) were synthesized and cloned into *pUAST-6xMyc* Gateway vector using similar protocols above for S2 cell expression and Co-IP analysis.

For Western blot, protein samples were extracted from 3rd instar larvae and analyzed by standard biochemical procedures. Primary antibodies used include: anti-α-Tubulin (mouse, Sigma B5-1-2, 1:500000), anti-Flag (rabbit, Sigma F7425, 1:1000), anti-Cnx (gift from Nansi Colley, 1:1000), and anti-Para (made in this study, mouse, 1:250).

For RT-PCR, total RNAs were extracted from fly adults with Trizol and reversely transcribed into cDNA using the QuantScript RT Kit (Tiangen Biotech). Primers sequences are as follows:

*cnx99A* 5′ primer 5′-AATCGGACTCATTCGTG-3′
*cnx99A* 3′ primer 5′-CGAAACGAGTGTGTGTGTGGTGTG-3′
*cnx11A* 5′ primer 5′-GGCTCCAATGGTAGACAA-3′
*cnx11A* 3′ primer 5′-AATGCCCGCTTACTCCTC-3′
*cnx14D* 5′ primer 5′-ATTGTCATAGTGGCCATTGCG-3′
*cnx14D* 3′ primer 5′-ATCATTTGGTTGTTGGTGCTCTG-3′

For Co-IP analysis, protein lysates from S2 cells or adult heads were harvested and lysed using the standard protocols. Para antibody was first conjugated to protein A/G agarose (sc-2003, Santa Cruz) for 2–2.5 h at 4°C. After serial washes with lysis buffer, resins were subjected to incubation with protein lysates from S2 cells or adult heads for another 2–2.5 h at 4°C. After incubation and subsequent washes, the final eluate was subjected to SDS-PAGE analysis.

### Generation of para antibody

Three Para antibodies have been generated in this study. The peptide IHSRSPSITSRTADV (2,117–2,131) at the C-terminus of Para protein was first used to generate a polyclonal antiserum in guinea pigs against Para. Due to poor staining results, proteins made from the Para N-terminal amino acid 1–149 was generated to make polyclonal antiserum against Para in both rabbits and mice (ABclonal, China). In the present study, mouse anti-Para antibody generated against N-terminal amino acid 1–149 was used throughout the study.

### Electrophysiology

Third instar larvae were carefully dissected as described previously (Jan and Jan, 1976). Briefly, wandering third instar larvae were dissected in Ca^2+^-free HL3.1 saline, gut and fat were removed, and the body wall was spread out to expose the nerve and muscle. For excitatory junction potential (EJP) recordings, the segmental nerve was cut, and the free end containing all blunt nerves was drawn into a microelectrode and stimulated with a Grass S48 stimulator (Astro-Grass) at 0.3 Hz with suprathreshold stimulating pulse. Twenty-five to thirty EJPs in NMJ muscle 6 of A3 segment were recorded using the recording electrodes (20–50 MΩ) filled with 3 M KCl. Miniature EJPs (mEJPs) were recorded for 8 s after EJP recordings. All recordings were conducted in modified HL3.1 solution containing 70 mM NaCl, 5 mM KCl, 4 mM MgCl2, 10 mM NaHCO3, 0.6 mM CaCl2, 115 mM sucrose, 5 mM trehalose, and 5 mM HEPES, pH 7.2. Recordings were performed at room temperature with an Axoclamp 900A amplifier (Molecular Devices, Sunnyvale, CA) in bridge mode. Recording data were digitized with a Digitizer 1322A (Molecular Devices) and analyzed by the Clampfit 10.2 software. At least 7 NMJs were analyzed and three independent experiments were performed for each genotype.

To measure latency time (the time from the end of stimulation to the beginning of the response, usually about 1–2 ms), Clampfit 10.2 software was used to analyze collected raw data on electrophysiological parameters such as EJPs and mEJPs. Similar lengths of stimulated nerves were analyzed for all samples. By pointing the cursors to the end of stimulation and the beginning of baseline deflection, the latency time in between was recorded by the software. These values were then averaged and plotted as graphs.

### Immunohistochemistry and microscopy

NMJs from third instar larvae and adult brains from flies of 1 week old without mouthparts were dissected and fixed with 4% formaldehyde in 1X PBS following standard protocols previously described. Reagents used include: Normal Donkey Serum (0.5%, NDS, Jackson Lab), mouse anti-Repo (1:100, DSHB), rat anti-Elav (1:500, DSHB), rabbit anti-Cnx (1:500, gift from Nansi Colley), mouse anti-Para (1:100, made in this study), anti-HRP-TRITC (1:500, Jackson Lab), and anti-HRP-Cy5 (1:500, Jackson Lab). All other secondary antibodies were purchased from Jackson Lab. Images were captured with Leica TCS SP5 confocal microscopy. Details on brain dissection, immunostaining, image acquisition, and quantification of fluorescent intensities were included in the supplementary materials.

### Behavioral analysis

Rapid iterative negative geotaxis (RING) assay using a multi-cylinder electrical system and analysis software RflyDetection were developed with help from Fude Huang (SARI Center for Stem Cell and NanoMedicine, Shanghai Advanced Research Institute, CAS) and according to previous protocols (Gargano et al., 2005; Liu et al., 2015). Ten cylinders (inner diameter: 20 mm, height: 140 mm) were placed side by side and each contains at least 10 unisex flie at least 90 flies were analyzed per genotype per experiment and three independent experiments were performed. To analyze the climbing ability, an initial mechanical shock was applied so that all flies were tapped down to the bottom of the cylinder and synchronized. Climbing distances were calculated with help from the RflyDetection software (allows automatic detection of the fly position within the cylinder) by analyzing video images captured every 7 s from recording (Sony digital camera, HDR-CX220E). Similar synchronized action was repeated several times and climbing distance for each fly was measured and averaged. Flies of 3 days old were collected after CO_2_ anesthesia and placed in vials with fly food (no yeast) for 1 day before transferring to the cylinders for experiments.

To record the recovery time from paralysis, 5 flies in vials were first put in a 38°C water bath for 25 s, allowing all flies became completely paralyzed, then shifted to the room temperature. Total about 100 flies were analyzed for each genotype. The recovery time was recorded from the moment the vials were taken out of the water bath to the time flies are free to walk and move on the feet. The same vials were used throughout the experiments. Then, recovery time was transformed into frequency data in an interval of 10 s. The percentage of recovered over total number of flies (Recovered/Total flies, %) was plotted against time at an interval of 10 s from 0 to 120 s using SPSS and Prism software. *P*-values of significance at each time point were calculated with 4-fold table Chi-Square Test (Fisher's Exact Test) and indicated with asterisks. ^*^p < 0.05, ^**^p < 0.01, ^***^p < 0.001.

### Statistical analysis

Intensities of fluorescence or GFP were measured and quantified using the Leica confocal software. Step-by-step protocols with snapshots of images were included in the supporting materials. For adult brains, three regions of equal area were chosen bilaterally for the measurement. For VNC axons, axons proximal, or distal to VNC in segment A2 or A4-5 were chosen and quantified. *P*-values of significance (indicated with asterisks, ^*^p < 0.05, ^**^p < 0.01, ^***^p < 0.001) were calculated using unpaired *T*-test between two groups and one-way ANOVA with Bonferroni multiple comparison test among three groups or above. ns means no significance.

## Results

### Cnx and para colocalize in neuronal ERs

To analyze Cnx endogenous expression in the nervous system, adult brains were first co-stained with antibodies against Cnx (Rosenbaum et al., 2006) and Embryonic lethal, Abnormal vision (Elav) or Reversed polarity (Repo), widely used markers for labeling neurons and glia respectively (Robinow and White, 1991; Xiong et al., 1994; Figures S1A–D”). Our results suggest that Cnx localizes around the neuronal nuclei labeled by Elav, whereas no significant overlapping pattern was observed for Cnx and the glial nucleus marker Repo (Figures S1B”,D”). Next, Para protein expression was analyzed using antibodies raised against the N-terminal amino acid sequence 1–149 in this study (please see Materials and Methods for details on the antibody). Similar to Cnx, Para proteins were detected around the Elav-positive nuclei in adult brains co-stained with Para and Elav (Figures S1E–F”). On the other hand, no significant overlapping pattern was detected for Para and GFP in the adult brains expressing *UAS-mCD8-GFP* under control of the pan-glial *Repo-Gal4* (*Repo*>*mCD8-GFP*, Figures S1G–H”).

**Figure 1:**
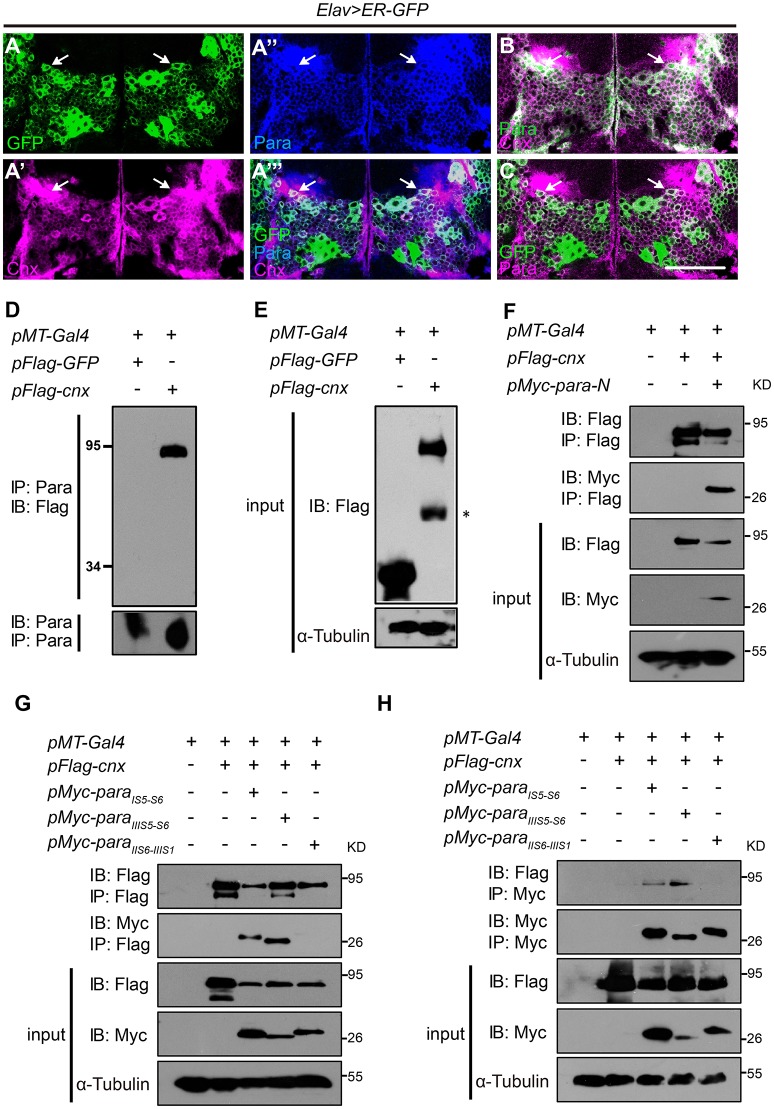
**Cnx colocalizes and interacts with Para. (A–C)** Adult brains carrying *Elav*>*ER-GFP* were co-stained with Cnx (magenta) and Para (blue in **A”** and **A”’**, green in **B**, or magenta in **C**). Note that most of the GFP-positive cells were also Cnx- and Para- positive (white arrows in **A”’** and **C**) and that Cnx and Para were colocalized (white arrows in **A”’,B**). Scale bar: 50 μm. **(D,E)** Protein lysates from S2 cells transfected with *3xFlag-GFP* (control) or *3xFlag-cnx* plasmids were subjected to Co-IP analysis. Our results revealed that Para interacts with Cnx and they are in the same protein complex. ^*^ non-specific band. **(F)** Co-IP analysis revealed that Cnx interacts with the first Para N-terminal intracellular segment (Para-N, amino acid 1–149). **(G,H)** Co-IP analysis on the interaction between Cnx and three Para protein variants: 6xMyc-Para_IS5-*S*6_, 6xMyc-Para_IIIS5-*S*6_, or 6xMyc-Para_IIS6-*IIIS*1_. Proteins sizes of three para variants were about 25–30 kDa shown on the blots. Note that Cnx interacts with the two Para fragments containing glycosylation sites (6xMyc-Para_IS5-*S*6_ and 6xMyc-Para_IIIS5-*S*6_), but not the variant without one. Co-IP analyses were done by both anti-Flag- or anti-Myc-antibody conjugated beads.

To further analyze the localization pattern, transgenic flies carrying *UAS-GFP.KDEL* were used to express GFP with a C terminal endoplasmic reticulum (ER) retention signal under control of the pan-neuronal *Elav-Gal4* (*Elav*>*ER-GFP*). These flies exhibit GFP signals that label ER in neurons and were analyzed for their GFP colocalization with Cnx or Para. Majority of Cnx was found to exhibit overlapping signals with *Elav*>*ER-GFP*, suggesting that Cnx localizes in neuronal ERs (white arrows in Figures S1J–J”). Para also exhibited a similar pattern and largely colocalized with ER-GFP (white arrows in Figures S1L–L”). Images in higher resolution of *Elav*>*ER-GFP* adult brains co-stained with Cnx and Para showed strong overlapping signals for Cnx, Para, and GFP (white arrows in Figures 1A–C). Cnx and Para also colocalized in the adult eye (data not shown), consistent with observations from previous study on Cnx functions in rhodopsin maturation. Interestingly, immunostaining of larval ventral nerve cord (VNC) from *Elav*>*ER-GFP* flies also showed colocalization of Cnx, Para, and neuronal ERs (Figure S2). Taken together, these results suggest that Cnx colocalizes with Para and both proteins exhibit an ER pattern in *Drosophila* adult neurons.

### Cnx interacts with para

Based on the colocalization pattern, we then sought to determine whether Cnx interacts with Para and exists in the same biochemical complex. First, the full-length *cnx* or the control *GFP* was cloned into plasmids carrying a N-terminal Flag epitope in three tandem repeats (3xFlag) for expression in S2 cells. Protein lysates transfected with *3xFlag-GFP* or *3xFlag-cnx* were subjected to Co-IP analysis using resins conjugated with the Para antibody. After pull down, the final eluate was analyzed by SDS-PAGE for the presence of Cnx proteins. Strikingly, Cnx was detected in the eluate of S2 cells transfected with *3xFlag-cnx* using the Flag antibody, whereas no GFP proteins were detected in the control (Figures 1D,E). These results suggest that Cnx interacts with Para and they are in the same protein complex. Furthermore, endogenous protein lysates from wild-type adult heads were collected and subjected to Co-IP analysis using the Para-conjugated resins. Our analysis also revealed a strong interaction between endogenous Para and Cnx by detecting the presence of Cnx proteins in the eluate (Figure S3). As a control, Cnx was not detected in the resins without Para conjugation. Specificity of the Para antibody was verified by Western blot analysis and immunostaining using a *para* RNAi fly line (Parker et al., 2011b) (*paraR*^*6131*^, Figure S4). Western blot results indicate a decrease in Para protein levels detected by the antibody upon RNAi expression in neurons, suggesting that the newly generated Para antibody is specific (Figure S4A). Whereas, a reduction in *para* mRNA levels was detected by RT-PCR (Figure S4B), immunostaining experiments using adult brains (Figures S4C–G) and VNC motor axons (Figure S4H) also suggested that Para protein levels detected by our antibody were significantly reduced upon RNAi expression.

Due to the fact that Para is a large transmembrane protein (>200 kDa), and that we were unable to obtain a Para cDNA clone thus far, we synthesized and cloned the first N-terminal intracellular domain (Para-N, before domain I segment 1, amino acids 1–149) and tested whether it interacts with Cnx. Consistent with previous results, our Co-IP analysis indicates that Para-N proteins were detected in the Cnx eluates, confirming an interaction between Cnx and Para (Figure 1F).

### Cnx-para interaction requires para glycosylation sites

Our confirmation on the ER localization of Cnx and Para, their interaction, and the glycosylation nature of sodium channel proteins (Mercier et al., 2015; Shabbir et al., 2015) suggests that Para could well be a substrate under Cnx quality control. We then looked for potential glycosylation sites (Glc_1_Man_9_GlcNAc_2_ of amino acid Asn at a consensus sequence Asn-X-Ser/Thr) on Para and studied whether Cnx interacts with Para on these sites. Uniprot prediction analysis uncovered similar glycosylation sites on Para and its human homolog SCN2A (unpublished observations, Figure S5). We selectively synthesized two Para protein fragments that contain predicted glycosylation sites between Para domain I segment 5 and 6 (Para_*IS*5−*S*6_) or Para III domain segment 5 and 6 (Para_IIIS5−S6_). On the other hand, Para protein fragment between Para domain II and III (Para_IIS6−IIIS1_) that contains no predicted glycosylation sites was also synthesized to serve as a negative control. Para_*IS*5−*S*6_ contains three predicted glycosylation sites at Asn313, 325, and 343, whereas Para_IIIS5−S6_ contains two at Asn1463 and 1482. These proteins were all tagged with 6xMyc epitopes with sizes about 25–30 kDa shown on the blots.

To analyze if Cnx binds to any of these Para protein variants, 6xMyc-Para_*IS*5−*S*6_, 6xMyc-Para_IIIS5−S6_, or 6xMyc-Para_IIS6−IIIS1_ was co-transfected with Cnx in S2 cells and co-immunoprecipitated. Interestingly, Co-IP analysis showed that not only Cnx proteins were detected in the pull down of Flag beads, the two Para protein variants that contain potential glycosylation sites, Para_*IS*5−*S*6_ and Para_IIIS5−S6_, were also detected in the eluates (Figures 1G,H). On the other hand, Para_IIS6−IIIS1_ that contains no predicted glycosylation sites was not co-immunoprecipiated by Cnx. These results suggest that Cnx interacts with Para and their interaction is likely dependent on glycosylation sites within these fragments.

### Downregulation of Cnx expression causes a reduction in para protein levels

Based on above biochemical results, we then sought to determine if Cnx regulates Para protein levels. Two RNA interference (RNAi) lines, *cnxR*^*100740*^, and *cnxR*^*42397*^, from the Vienna *Drosophila* RNAi Center (VDRC) were used after verification of their efficiencies by RT-PCR and Western blot analysis (Figures S6A,B). Furthermore, transgenic flies that overexpress Cnx (*UAS-3xFlag-cnx*^*5-2*^ or *UAS-3xFlag-cnx*^*3-1*^, abbreviated as *cnx*^*5-2*^ or *cnx*^*3-1*^) were constructed and Cnx protein levels in these lines were verified using Western blot analysis (Figure S6C). In these analyses, both RNAi lines abolish Cnx expression with similar efficiencies. Cnx expression levels were also similar upon expressing either transgenic lines (Figure S6). In order to be more comprehensive and easier to follow for the readers, a list of flies used in the current study was described in Table S1. In addition, data from flies expressing either *cnx* RNAi and/or *cnx*^*5-2*^ will be presented in the following result figures.

**Figure 2:**
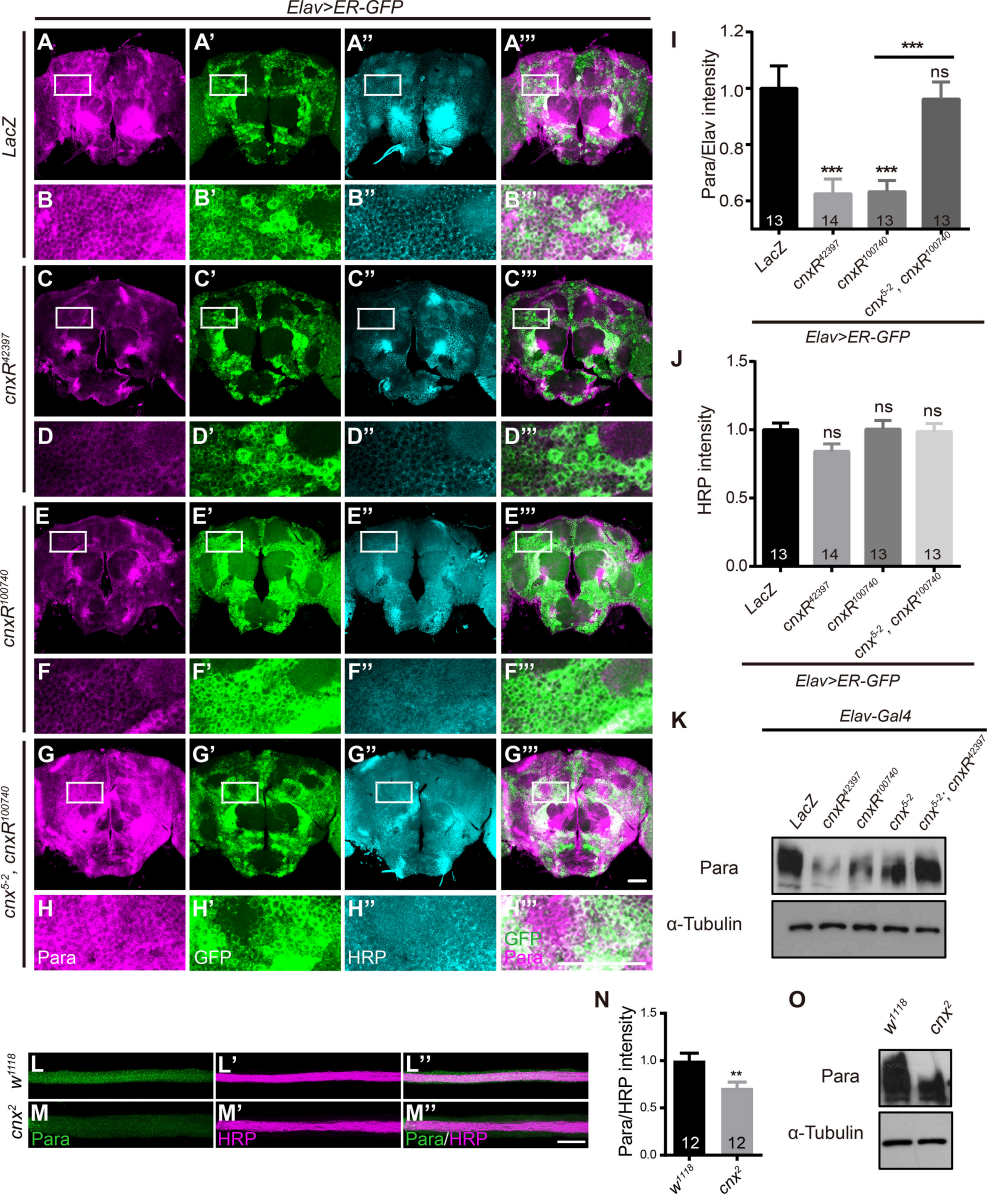
**Para protein levels are reduced when down regulating Cnx expression. (A–H”’)** Adult brains expressing *Elav*>*ER-GFP* were dissected and stained with Para (Magenta) and HRP (Cyan). White rectangles represent areas enlarged and shown directly underneath. Genotypes shown: *Elav; ER-GFP*>*LacZ* **(A–B”’)**, *Elav; ER-GFP*>*cnxR*^*42397*^ **(C–D”’)**, *Elav; ER-GFP*>*cnxR*^*100740*^ **(E–F”’)**, and *Elav; ER-GFP*>*cnx*^*5-2*^, *cnxR*^*100740*^ (**G–H”’)**. **(I,J)** Statistics for the intensities of Para/HRP **(I)** and HRP **(J)** in adult brains. Note a significant decrease in Para/HRP intensities when *cnxR*^*42397*^ or *cnxR*^*100740*^ was expressed. **(K)** Western blot analysis on Para protein levels in adult brains (*n* = 50) carrying the following genotypes: *Elav*>*LacZ, Elav*>*cnxR*^*42397*^, *Elav*>*cnxR*^*100740*^, *Elav*>*cnx*^*5-2*^*, Elav*>*cnx*^*5-2*^*; cnxR*^*42397*^. Input control: α-Tubulin. **(L–M”)** VNC motor axons were stained with Para (Green) and HRP (Magenta) in *w*^*1118*^ and *cnx*^*2*^ larval NMJs. **(N)** Statistics for the intensities of Para/HRP in *w*^*1118*^ and *cnx*^*2*^ axons. Note a decrease in Para levels in *cnx*^*2*^ axons. **(O)** Western blot analysis revealed a decrease in Para protein levels in *cnx*^*2*^ mutant adult brains (*n* = 50). Input control: α-Tubulin. Data were shown as mean ± SEM. Scale bar: 20 μm (NMJs) and 50 μm (adult brains). Number of adult brains dissected per genotype was shown in the bars. At least 13 brains were analyzed per genotype and six different areas in the brains were selected. ^**^p < 0.01, ^***^p < 0.001. Data were shown as mean ± SEM. At least three independent experiments were performed.

Using these tools and the newly generated Para antibody, we were able to assess whether manipulation of Cnx expression leads to a change in Para expression, thus a change in its activity and function. To address this, we first examined Para protein levels by immunostaining in *Drosophila* adult brains. Interestingly, Para protein levels, measured as the staining intensities of Para normalized to the membrane-targeted Horseradish Peroxidase (HRP) (Para/HRP), exhibited a strong decrease when Cnx expression was downregulated (*Elav; ER-GFP*> *cnxR*^*42397*^ or *cnxR*^*100740*^, 0.6 ± 0.1 or 0.6 ± 0.0 vs. 1.0 ± 0.1, *p* < 0.001, compare Figures 2D,F to Figures 2B,I). In the presence of *cnx*^*5-2*^ overexpression, the reduction in Para protein levels was restored (*Elav; ER-GFP*>*cnx*^*5-2*^*, cnxR*^*100740*^, 1.0 ± 0.1 vs. 0.6 ± 0.0, *p* < 0.001, compare Figure 2H to Figures 2F,I). Quantification of fluorescent intensities indicated that no significant difference in HRP intensities among all samples (Figure 2J). Furthermore, Western blot analysis revealed that Para protein levels were reduced in fly adult brains expressing either *cnx* RNAi (Figure 2K). Similar to our immunostaining results, Para protein levels analyzed by Western blot analysis were restored when Cnx and *cnx* RNAi were expressed together (Figure 2K). On the other hand, analysis of motor axons targeting out from the ventral nerve cord (VNC) indicated similar results. Para protein levels, also measured as the staining intensities of Para normalized to HRP, exhibited a significant decrease in axons of a *cnx* mutant, *cnx*^*2*^ (Rosenbaum et al., 2006; 0.7 ± 0.1 vs. 1.0 ± 0.1, *p* < 0.01, Figures 2L–N). Quantifications of HRP staining intensities indicated that protein levels of HRP in these tissues were similar among different samples and served as internal controls (data not shown). Western blot analysis also revealed a decrease in Para protein levels in *cnx*^*2*^ mutant flies (Figure 2O). Taken together, these results suggest that Cnx regulates Para expression and Para protein levels are reduced when Cnx expression is downregulated.

### Para protein levels are upregulated when overexpressing Cnx cytosolic calcium-binding domain

**Figure 3:**
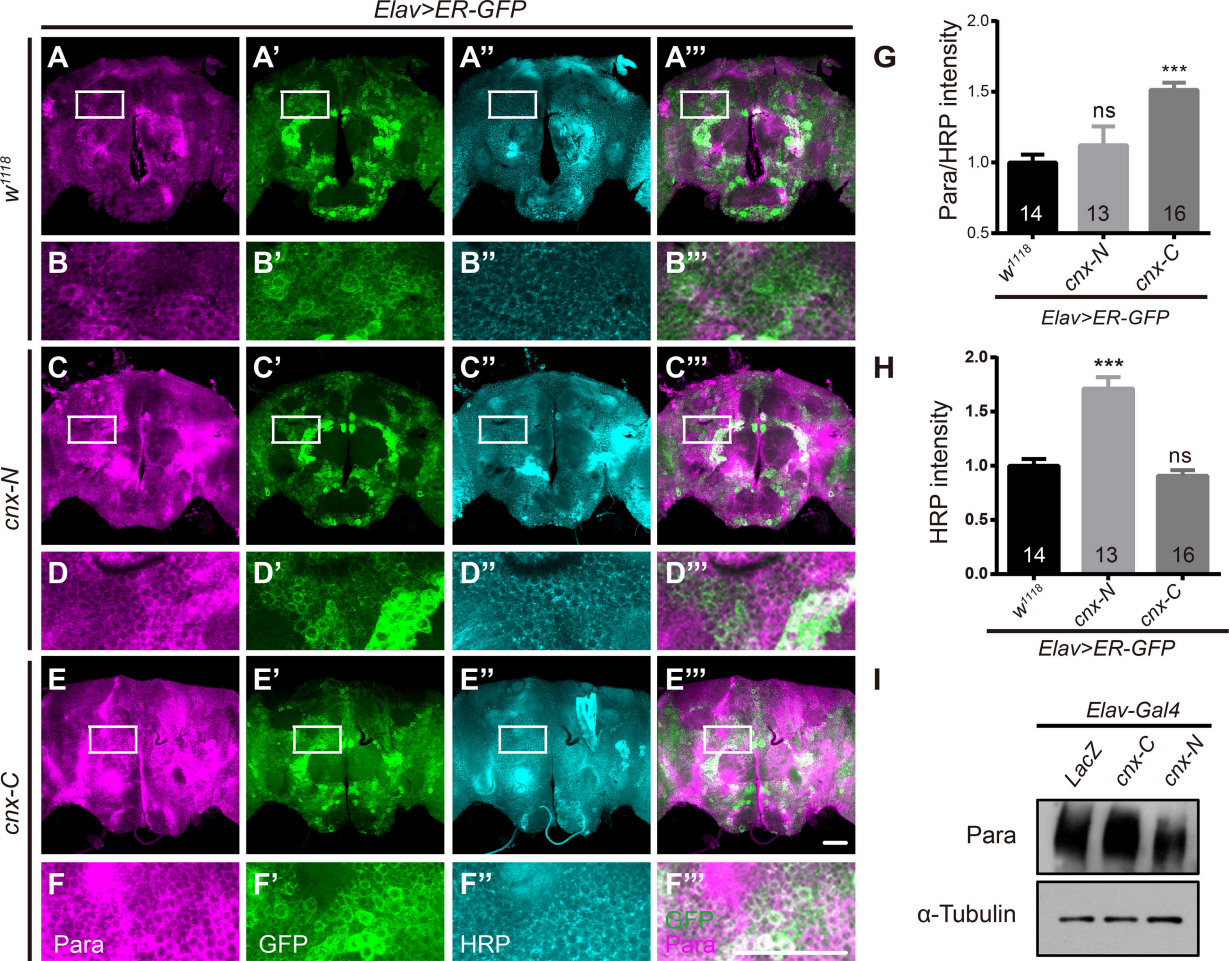
**Para protein levels are upregulated when overexpressing the Cnx C-terminal calcium-binding domain. (A–F”’)** *Elav*>*ER-GFP* adult brains expressing *LacZ, cnx-N*, or *cnx-C* were dissected and stained with Para (Magenta) and HRP (Cyan). Scale bar: 50 μm. White rectangles represent areas enlarged and shown directly underneath. **(G,H)** Quantification of Para/HRP intensities **(G)** or HRP **(H)** for the above genotypes. Note a significant increase in the Para/HRP intensities when Cnx-C was overexpressed. **(I)** Western blot analysis revealed an increase in Para protein levels when Cnx-C was overexpressed. ^***^p < 0.001. Data were shown as mean ± SEM. Number of adult brains dissected per genotype was shown in the bars. At least 13 brains were analyzed per genotype and six different areas in the brains were selected. At least three independent experiments were performed.

To further dissect the part of Cnx required for regulating Para function, truncated constructs of Cnx carrying only the N-terminal luminal domain (1–489aa, Cnx-N) or the C-terminal cytosolic domain (509–605aa, Cnx-C) were generated (Please see Materials and Methods for details). Para protein levels were analyzed in *Elav*>*ER-GFP* adult brains expressing Cnx-N or Cnx-C. Interestingly, a significant increase in Para/HRP intensities was detected when Cnx-C was overexpressed (*Elav; ER-GFP*>*cnx-C*, 1.5 ± 0.1 vs. 1.0 ± 0.1, *p* < 0.001, compare Figure 3F to Figures 3B,G). Quantifications of HRP intensities unexpectedly revealed a significant increase in Cnx-N overexpression, but not Cnx-C (Figure 3H). In addition, Western blot analysis indicated that Cnx-C overexpression caused an increase in Para protein levels in fly adult brains (Figure 3I). Interestingly, Co-IP analysis indicated that Cnx-C failed to interact with Para protein variants containing glycosylation sites (6xMyc-Para_IS5-*S*6_ and 6xMyc-Para_IIIS5-*S*6_), suggesting that Cnx C-terminal domain regulates Para activity independently of glycosylation (Figure S7). Taken together, these results suggest that Cnx cytosolic domain, with its calcium buffering ability, plays pivotal roles in regulating Para protein levels.

### Altered Cnx expression in neurons leads to fly locomotor deficits

**Figure 4:**
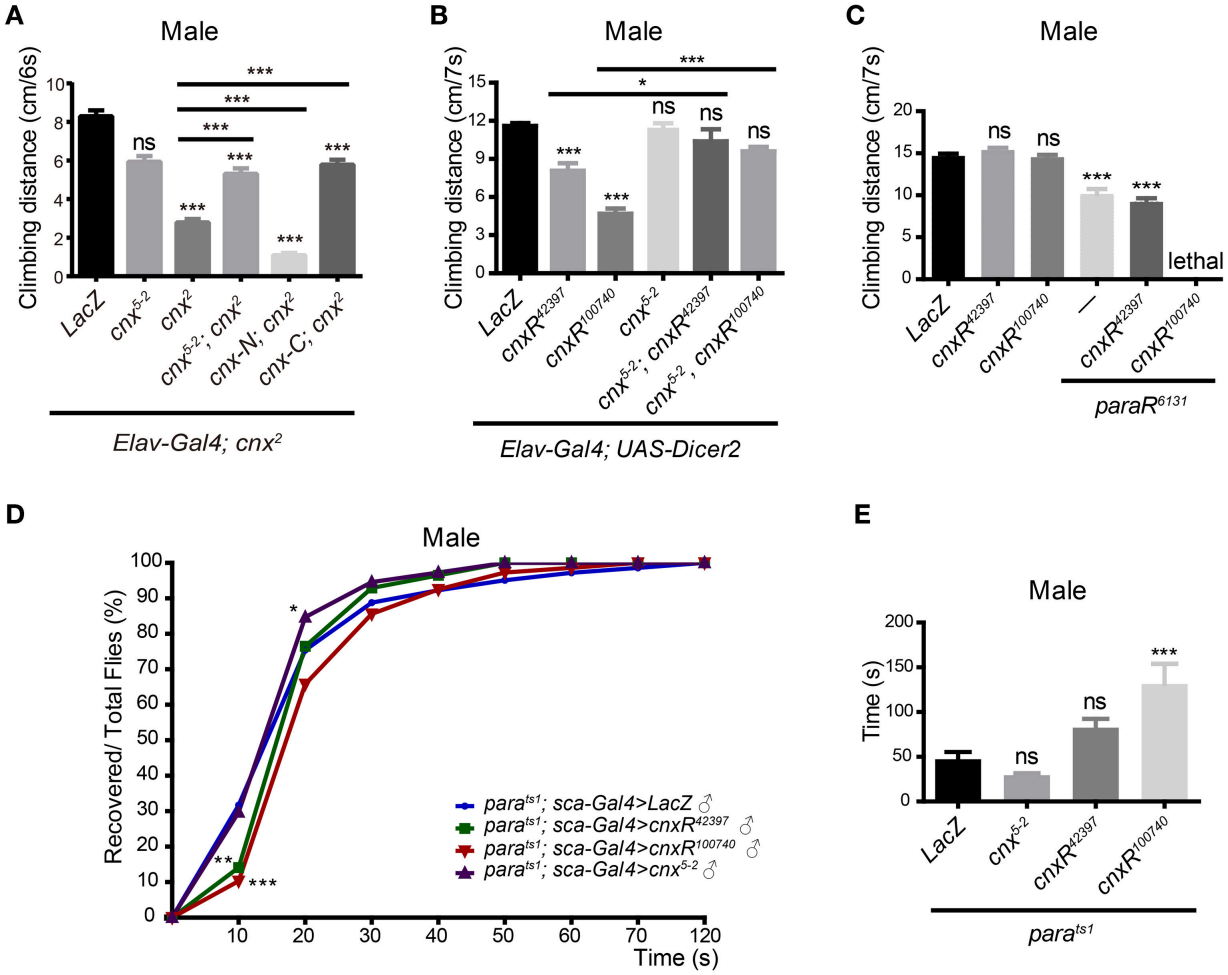
**Cnx regulates Para-mediated adult climbing and paralysis**. Adult climbing ability was analyzed by a multi-cylinder electrical system that allows synchronization of both time and activity. *w*^*1118*^ or *LacZ* was used as a control, and the climbing distance was measured at a time interval of 6–7 s. At least 90 flies were analyzed, with 10 in each cylinder, and three independent experiments were performed. Climbing distance for each genotype was plotted as bar graphs. Only male flies were shown. **(A)** Climbing distance was measured for flies carrying the following genotypes listed from left to right: *Elav; cnx*^2^>*LacZ, Elav; cnx*^*2*^>*cnx*^*5-2*^, *Elav; cnx*^*2*^>*cnx*^*2*^, *Elav; cnx*^*2*^>*cnx*^*5-2*^*; cnx*^*2*^, *Elav; cnx*^*2*^>*cnx-N; cnx*^*2*^, *Elav; cnx*^2^>*cnx-C; cnx*^2^. Note a significant decrease in climbing activity in *cnx*^*2*^ mutant (third gray bar from the left). Overexpression of full-length Cnx (*cnx*^*5-2*^) or Cnx containing only its C-terminus (*cnx-C*) rescued the reduction in climbing activity in *cnx*^*2*^ mutant, whereas overexpression of Cnx containing only its N-terminus (*cnx-N*) failed to do so. **(B)** Climbing distance was measured for flies carrying the following genotypes listed from left to right: *Elav; Dicer2*>*LacZ, Elav; Dicer2*>*cnxR*^*42397*^, *Elav; Dicer2*>*cnxR*^*100740*^, *Elav; Dicer2*>*cnx*^*5-2*^, *Elav; Dicer2*>*cnx*^*5-2*^*; cnxR*^*42397*^, *Elav; Dicer2*>*cnx*^*5-2*^*; cnxR*^*100740*^. *cnx* RNAi expression driven by *Elav; Dicer2* leads to a decrease in climbing distance (second and third gray bars from the left), whereas Cnx overexpression does not alter climbing ability. **(C)** Adult climbing ability was assessed similarly for the following genotypes: *Elav*>*LacZ, Elav*>*cnxR*^*42397*^, *Elav*>*cnxR*^*100740*^, *Elav*>*paraR*^*6131*^, *Elav*>*cnxR*^*42397*^*, paraR*^*6131*^, and *Elav*>*cnxR*^*100740*^*; paraR*^*6131*^. **(D)** Cnx regulates the recovery kinetic of Para-mediated paralysis. Percentage of recovered flies from paralysis (Recovered/Total flies, %) was plotted against time cumulatively at an interval of 10 s using SPSS and Prism software. Purple curves: *para*^*ts1*^*; Sca*>*cnx*^*5-2*^, blue curves: *para*^*ts1*^*; Sca*>*LacZ*, green curves: *para*^*ts1*^*; Sca*>*cnxR*^*42397*^, red curves: *para*^*ts1*^*; Sca*>*cnxR*^*100740*^. **(E)** Recovery time was defined differently as the time required for half of the flies recovered, climbed up, and passed the fixed height (4 cm). The time was calculated and plotted for the following genotypes: *para*^*ts1*^*; Sca*>*LacZ, para*^*ts1*^*; Sca*>*cnx*^*5-2*^, *para*^*ts1*^*; Sca*>*cnxR*^*42397*^, and *para*^*ts1*^*; Sca*>*cnxR*^*100740*^, ^*^p < 0.05, ^**^p < 0.01, ^***^p < 0.001, Data were shown as mean ± SEM.

Due to the fact that Para dysfunction has been shown to cause paralysis in flies, we examined whether Cnx, a factor that regulates Para expression, plays a role in controlling fly locomotor behavior. First, adult climbing ability was analyzed using the RING (rapid iterative negative geotaxis) assay (Gargano et al., 2005; Liu et al., 2015). A multi-cylinder electrical system and analysis software RflyDetection were developed according to previous protocols to score the climbing distance of individual fly (for details please see Materials and Methods). For simplicity, only results from males were shown (Figure 4). Results from females were included in Figure S8. In general, similar trends in the locomotor deficits were observed for both male and female flies. Taken advantage of this system, *cnx*^*2*^ mutant flies were first analyzed for possible climbing defects. Both male and female *cnx*^*2*^ flies exhibited a striking decrease in climbing distance compared to the wild-type control (2.8 ± 0.2 vs. 8.3 ± 0.3 in male flies, *p* < 0.001, Figure 4A; Figure S8A). Similar effects were seen when downregulating Cnx expression using *cnx* RNAi under control of the pan-neuronal *Elav-Gal4* with *Dicer2* (male flies: *Elav; Dicer2*>*cnxR*^*42397*^, 8.1 ± 0.6 vs. 11.6 ± 0.2, *p* < 0.001, *Elav; Dicer2*>*cnx*^*100740*^, 4.7 ± 0.4 vs. 11.6 ± 0.2, *p* < 0.001, Figure 4B; Figure S8B). On the other hand, neuronal Cnx overexpression using the transgenic flies *cnx*^*5-2*^ does not alter climbing ability (Figure 4B, Figure S8B). Despite so, simultaneous *cnx*^*5-2*^ expression rescued *cnx*^*2*^ mutant climbing defect in males (*Elav; cnx*^2^>*cnx*^*5-2*^*; cnx*^*2*^, 5.3 ± 0.3 vs. 2.8 ± 0.2, *p* < 0.001, Figure 4A). Furthermore, upon co-expression of *cnx*^*5-2*^, *Elav; Dicer2*>*cnx*^*42397*^ male flies exhibiting a locomotor defect have now climbed normally (*Elav; Dicer2*>*cnx*^*5-2*^*, cnxR*^*42397*^, 10.4 ± 0.9 vs. 8.1 ± 0.6, *p* < 0.05, Figure 4B). Similarly, *cnx*^*5-2*^ expression rescued climbing defects in both male and female *Elav; Dicer2*>*cnx*^*100740*^ flies (*Elav; Dicer2*>*cnx*^*5-2*^*, cnxR*^*100740*^, 9.6 ± 0.4 vs. 4.7 ± 0.4 in male flies, *p* < 0.001, 9.7 ± 0.3 vs. 8.0 ± 0.5 in female flies, *p* < 0.001, Figure 4B; Figure S8B). These results suggest that Cnx expression is required for adult climbing activity and lack of Cnx expression causes locomotor deficits.

In addition to the full length Cnx, Cnx-C overexpression rescued the *cnx*^*2*^ climbing defect in both male and female flies (*Elav;cnx*^*2*^ >*cnx-C;cnx*^*2*^, 5.8 ± 0.3 vs. 2.8 ± 0.2 in male flies, *p* < 0.001, 5.1 ± 0.6 vs. 2.8 ± 0.2 in female flies, *p* < 0.001, Figure 4A; Figure S8A), while Cnx-N overexpression failed to do so (Figure 4A, Figure S8A). These results suggest that N-terminal and C-terminal Cnx exhibit differential activity in regulating adult climbing. Whereas, the cytosolic domain containing the calcium-binding domain provides Cnx activity and rescues *cnx* mutant locomotor deficits, the N-terminal ER-luminal domain without the TM domain alone does not seem to provide sufficient function in regulating locomotor activity.

### Downregulation of Cnx expression exacerbates climbing deficits in *para* RNAi expressing neurons

Our results suggest that Cnx plays a role in controlling fly locomotor behavior. Next, we investigate whether Para, already known to cause paralysis, participates in the similar process. Our initial attempt of expressing *paraR*^*6131*^ under control of *Elav; Dicer2* failed as the driver is stronger than the pan-neuronal *Elav-Gal4* such that flies with abolished Para expression were dead. Instead, using *Elav-Gal4* to express *paraR*^*6131*^, a significant decrease in the climbing distance was detected (7.5 ± 0.5 vs. 10.8 ± 0.2 in male flies, *p* < 0.001, 7.5 ± 0.2 vs. 9.5 ± 0.2 in female flies, *p* < 0.001, Figure 4C; Figure S8C). Interestingly, in the presence of either *cnxR*^*100740*^ or *cnxR*^*42397*^, adult climbing distance was significantly reduced compared to downregulation of Para expression alone (5.6 ± 0.3 vs. 7.5 ± 0.2 in *Elav*>*cnxR*^*100740*^*; paraR*^*6131*^ female flies, *p* < 0.001, 6.5 ± 0.2 vs. 7.5 ± 0.2 in *Elav*>*cnxR*^*42397*^*, paraR*^*6131*^ female flies, *p* < 0.001, Figure S8C). In male flies, a similar reduction was detected for flies carrying *Elav*>*cnxR*^*42397*^*, paraR*^*6131*^, and simultaneous expression of *cnxR*^*100740*^ and *paraR*^*6131*^ in male flies resulted in lethality (Figure 4C). Based on these results, we conclude that Cnx genetically interacts with Para and Cnx-mediated Para expression is required for *Drosophila* adult climbing behavior.

### Cnx regulates the recovery kinetic of para-mediated paralysis

To further elucidate the importance of Cnx-mediated Para expression during fly behavioral control, a second type of behavioral paradigm, seizure-induced paralysis, was employed. Paralysis is classified as a type of behavior when the adult flies are flipped upside down with motionless legs and is detected in the loss-of-function *para*^*ts1*^ mutant upon temperature elevation (Suzuki et al., 1971; Siddiqi and Benzer, 1976; Wu and Ganetzky, 1980). Interestingly, *para*^*ts1*^–mediated paralysis is reversible and caused by a block in axonal conduction by electrophysiological studies (Wu and Ganetzky, 1980). Upon recovery, adult flies escape from paralysis and resume their normal position in walking and climbing. We took advantage of the *para*^*ts1*^ mutant and analyzed the effect of Cnx on Para-mediated paralysis. More than 100 *para*^*ts1*^ adult flies of each genotype were placed in separate vials and incubated at 38°C until completed paralysis. Then, the recovery time was recorded using a digital camera upon relocating the flies to room temperature (Video S1). At a time interval of 10 s, the number of recovered adult flies was scored and plotted against time cumulatively as percentage over the total number of flies. As shown in Figure 4D, Figure S8D, upon Cnx overexpression, a significantly higher percentage in the number of recovered adult flies was detected compared to the *para*^*ts1*^ control flies overexpressing *LacZ*, suggesting a shorter recovery time (purple curves in Figure 4D, Figure S8D, *p* < 0.05 for male flies, *p* < 0.01 for female flies, at 20 s). This piece of evidence suggests that neuronal-specific Cnx overexpression alters the recovery kinetic of *para*^*ts1*^ adult flies. On the contrary, reduced Cnx expression using either *cnx* RNAi significantly lowered the number of recovered *para*^*ts1*^ adult flies, suggesting a longer recovery time (green and red curves in Figure 4D, Figure S8D, *p* < 0.001 for either *cnx* RNAi, at 20 s). Bar graphs with SEM for each genotype were also plotted (Figure S9). In addition, the recovery time, defined differently as half of the flies recovered, climbed up, and passed the fixed height (4 cm), was plotted as bar graphs for each genotype (Figure 4E, Figure S8E). Similar results were obtained using this method, further supporting our conclusion. Expression of *cnx* RNAi has prolonged the recovery time (56.8 ± 6.7 vs. 36.2 ± 2.8 in *para*^*ts1*^*; Sca*>*cnxR*^*42397*^ female flies, *p* < 0.05, 76.3 ± 5.7 vs. 36.2 ± 2.8 in *para*^*ts1*^*; Sca*>*cnxR*^*100740*^ female flies, *p* < 0.001, Figure S8E). Altogether, these results suggest that altered Cnx expression in neurons greatly affects the recovery profile of *para*^*ts1*^ adult flies. Reduced Cnx expression prolongs the recovery time of *para*^*ts1*^ adult flies.

### Cnx promotes para-mediated ghost bouton formation during NMJ synaptogenesis

Neuronal activity that controls synapse formation and function lays the foundation for control over locomotor behavior. *Drosophila* larval neuromuscular junction (NMJ), with its unique genetic accessibility and similarity across species, is one of the most frequently used models in examining synaptogenesis pertaining growth, function, and behavior (Menon et al., 2013). Previously, we have shown that Para localizes on the motor axon targeted out from VNC (Figures 2L–N). As a sodium channel gene that regulates neuronal excitability, it is also likely that Para participates in NMJ synaptogenesis. To this end, we sought to determine whether Cnx-regulated Para expression regulates synaptogenesis, in attempt to understand the synaptic mechanism that underlies locomotor control.

**Figure 5:**
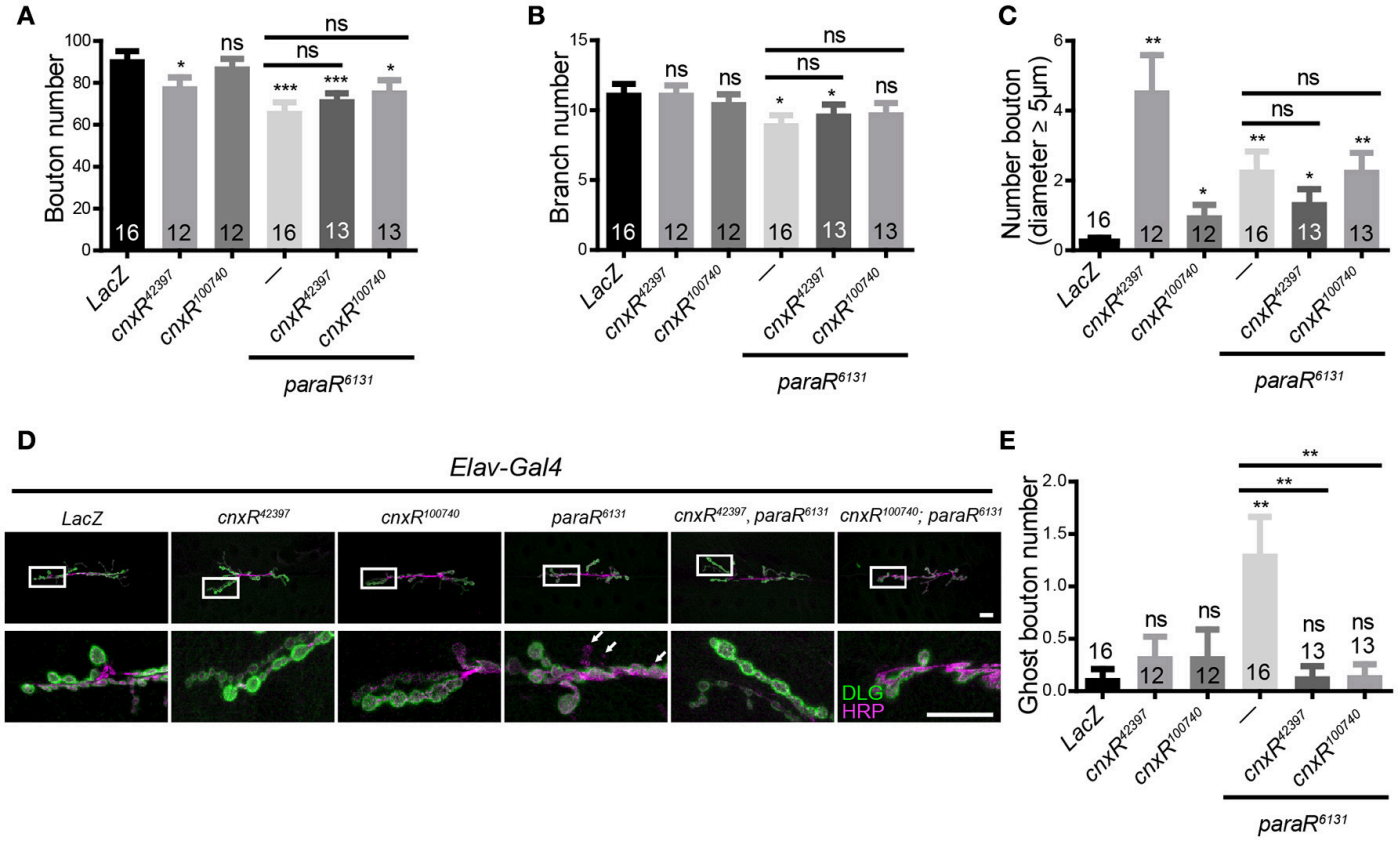
**Cnx regulates Para-mediated ghost bouton formation in NMJs**. Number of boutons **(A)**, branches **(B)**, and boutons with diameter > 5 μm **(C)** were quantified for muscle 6/7 NMJs. Results were shown for the following genotypes: *Elav*>*LacZ, Elav*>*cnxR*^*42397*^, *Elav*>*cnxR*^*100740*^, *Elav*>*paraR*^*6131*^, *Elav*>*cnxR*^*42397*^*, paraR*^*6131*^, and *Elav*>*cnxR*^*100740*^*; paraR*^*6131*^. **(D)** Muscle 6/7 NMJs stained with HRP (Magenta) and DLG (Green) were shown for the above genotypes. Note an increase in the number of ghost boutons stained positively for HRP but not DLG in *Elav*>*paraR*^*6131*^ NMJs (white arrows). **(E)** Quantification of ghost bouton numbers for NMJs carrying the above genotypes. Note a decrease in the number of ghost boutons in the presence of both *cnx* and *para* RNAi expression, ^*^p < 0.05, ^**^p < 0.01, ^***^p < 0.001, Scale bar: 20 μm. Data were shown as mean ± SEM. Number of NMJs per genotype was shown in the bars. At least 12 NMJs were analyzed and three independent experiments were performed for each genotype.

Our analysis of NMJ morphology at muscle 6/7 revealed that numbers for boutons, branches, and bigger-sized boutons with diameter over 5 μm were affected upon Para misexpression in neurons (Figure 5). Yet, these parameters were not rescued in the presence of either *cnx* RNAi (Figures 5A–C), suggesting that Para exhibits function independent of Cnx in NMJs. Interestingly, the number of ghost boutons, a type of boutons predominantly presynaptic and devoids of postsynaptic components (Koles and Budnik, 2012; Menon et al., 2013), was significantly increased when Para expression was downregulated (white arrows in Figure 5D, 1.3 ± 0.4 vs. 0.1 ± 0.1 in *Elav*>*paraR*^*6131*^ flies, *p* < 0.001, Figure 5E). Strikingly, the number of ghost boutons decreased upon co-expression of *para* and *cnx* RNAi, suggesting that Cnx expression affects Para-mediated ghost bouton formation (Figure 5D, 0.2 ± 0.1 vs. 1.3 ± 0.4 in *Elav*>*cnxR*^*100740*^*; paraR*^*6131*^ flies, *p* < 0.001, and 0.1 ± 0.1 vs. 1.3 ± 0.4 in *Elav*>*cnxR*^*42397*^*; paraR*^*6131*^ flies, *p* < 0.001, Figure 5E). Taken together, these results suggest that Cnx genetically interacts with Para to control the developmental dynamic of undifferentiated ghost boutons during the maturation of NMJ synapse formation (Figures 5D,E).

### Cnx and para affect the latency time and mEJP frequency during synaptic function

**Figure 6:**
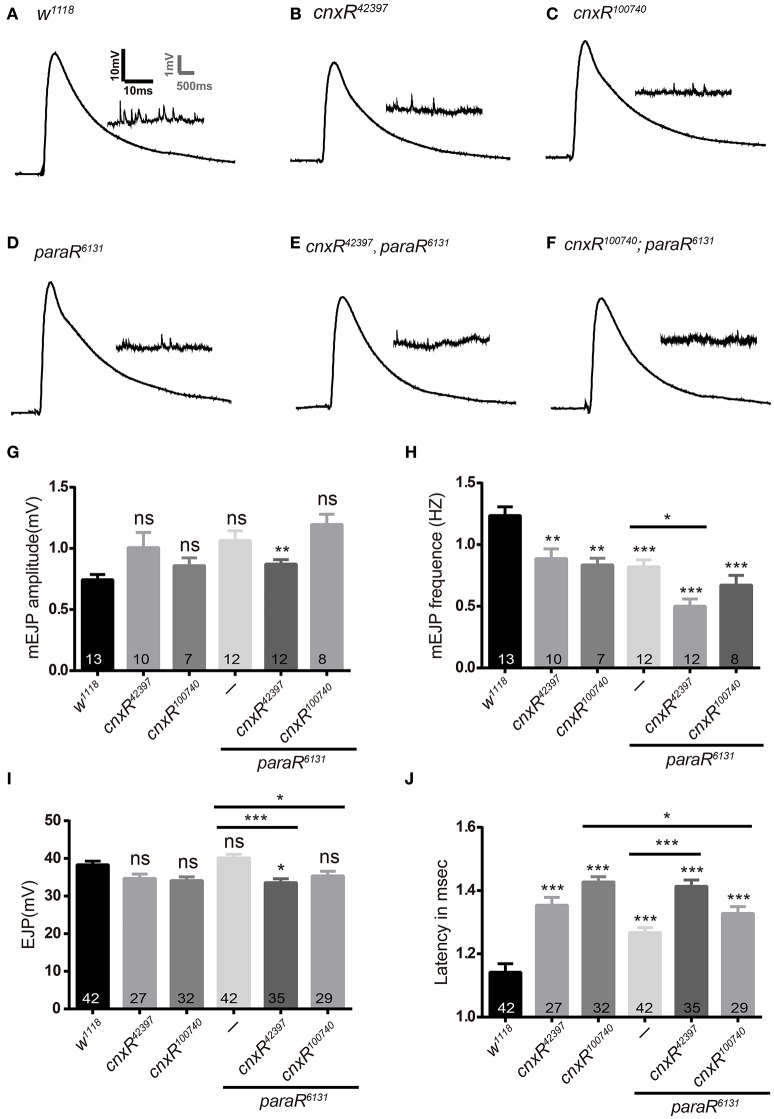
**Cnx and Para regulate the latency time and mEJP frequency during synaptic function. (A–F)** Samples traces of EJPs and mEJPs were shown for the following genotypes: *Elav*>*w*^*1118*^, *Elav*>*cnxR*^*42397*^, *Elav*>*cnxR*^100740^, *Elav*>*paraR*^*6131*^, *Elav*>*cnxR*^*42397*^*, paraR*^*6131*^, and *Elav*>*cnxR*^*100740*^*; paraR*^*6131*^. **(G–J)** Statistics for electrophysiological parameters such as mEJP amplitude (mV, G), mEJP frequency (Hz, H), EJP amplitude (mV, I), and latency time (msec, J) were shown. Note that mEJP frequency and the latency time were generally affected when Cnx or Para expression was downregulated. A further decrease in mEJP frequency **(H)** or increase in the latency time **(J)** was detected when both *cnx* and *para* RNAi were expressed. ^*^p < 0.05, ^**^p < 0.01, ^***^p < 0.001, Data were shown as mean ± SEM. At least 7 NMJs were analyzed and three independent experiments were performed for each genotype.

Since Para belongs to sodium channels that cluster on the axonal membrane and mediate ion influx for action potential propagation, it is intriguing to investigate further on how Cnx regulates Para activity by understanding whether Para-producing sodium currents are affected by Cnx expression. Our initial attempt to measure sodium currents failed as no noticeable sodium currents were detected in *Drosophila* S2 cells (unpublished observations). We then turned our attention to larval NMJs, a well-established *in-vivo* setting where we will be able to measure electrophysiological properties on altered Cnx and/or Para function. Our previous results have implicated a Cnx-mediated presynaptic modulation on Para activity at NMJs and further prompted us to analyze any possible defects during synaptic function correlating with the morphological difference that we detected. Using standard electrophysiology protocols, we determined NMJ electrophysiological parameters in the presence of *cnx* RNAi, *para* RNAi, or both. By expressing these RNAi using *OK6-Gal4* presynaptically, significant changes in mEJP frequency and latency time were detected, whereas the mEJP and EJP amplitudes were less affected (Figure 6). Sample traces and statistical results showed that downregulation of Para expression alone caused a significant increase in the latency of muscle response, suggesting that action potential propagates slower to the targeted synapse due to the absence of Para (1.3 ± 0.0 vs. 1.1 ± 0.0, *p* < 0.001, Figure 6J). The latency time then significantly increased when *cnx* RNAi was expressed together, suggesting that Cnx plays a role in modulating the latency time controlled by Para (1.3 ± 0.0 vs. 1.4 ± 0.0, *p* < 0.001, Figure 6J). On the other hand, mEJP frequencies were also affected in the absence of Para expression (0.8 ± 0.1 vs. 1.2 ± 0.1, *p* < 0.001, Figures 6D,H), and further reduced when both *para* and *cnx* RNAi were expressed (0.5 ± 0.1 vs. 0.8 ± 0.1, *Elav*>*cnxR*^*42397*^*; paraR*^*6131*^, *p* < 0.05, Figures 6D–F,H). Finally, the EJP amplitude was also significantly changed when *cnx* RNAi was co-expressed with *para* RNAi (33.6 ± 1.0 vs. 40.2 ± 0.9, *p* < 0.001, and 35.3 ± 1.3 vs. 40.2 ± 0.9, *p* < 0.05, Figures 6D–F,I). Taken together, these results suggest that Cnx modulates Para-controlled electrophysiological activity, in particular the latency time, mEJP frequency, and EJP amplitude.

## Discussion

### Cnx chaperone functions in the nervous system

Cnx is generally considered as a ubiquitous chaperone, yet its potential substrates and functions in the nervous system remain mysterious. In the past, studies addressing Cnx function in the nervous system have been limited to loss-of-function phenotypical analysis. Using a combination of approaches such as immunostaining, transgenic gene expression, and behavioral analysis, our study shows for the first time that Cnx localizes in neuronal ERs, plays a role in ER quality control, and is required for adult locomotor behavior. We also show that Cnx expression is precisely tuned in neurons for the control of locomotor activity. These observations uncover a critical role for Cnx in neurons, albeit a glial-mediated myelination defect has been reported for *cnx*-deficient mice (Denzel et al., 2002; Kraus et al., 2010). Intriguingly, no significant ensheathment phenotype was seen upon Cnx RNAi expression as exemplified in our study using the glial wrapping of embryonic peripheral nerves, a well-characterized model for studying glial ensheathment in *Drosophila* (data not shown; Banerjee and Bhat, 2008; Blauth et al., 2010). Due to a lack of subsequent myelination process in *Drosophila*, it is feasible to speculate that Cnx affects myelination at a later step but not the initial ensheathment process in both model systems.

Interestingly, our results assessing the role of Cnx during adult climbing activity has revealed a difference in function for Cnx N-terminus and C-terminus (Figure 4A). Expression of a truncated protein containing only the C-terminal Cnx consistently rescued the climbing defect in *cnx* mutant, suggesting that the calcium-binding domain at the Cnx C-terminus is crucial in regulating Cnx-controlled locomotor behavior. On the contrary, expression of a truncated protein containing the N-terminal Cnx (both globular lectin domain and P domain) failed to rescue the same climbing defect, suggesting that this part of Cnx does not provide sufficient activity to mediate locomotor activity.

### Para as a glycosylated substrate for Cnx in controlling locomotor behavior

Cnx is well-characterized for its function in nascent glycoprotein folding and ER retention (Williams, 2006; Caramelo and Parodi, 2008; Lederkremer, 2009; Määttänen et al., 2010; Benham, 2012). Despite the fact that Cnx is a general chaperone and regulates the stability of a number of substrates, our results and previous studies implicate that Para exhibits a role during Cnx-mediated locomotor behavior. Interestingly, sodium channels are heterotrimeric complexes composed of α, β1, and β2 subunits (Catterall, 1988, 1992; Tejedor and Catterall, 1988) and among them the α subunits have been shown to undergo glycosylation as matured from the biosynthetic precursor in rat brain neurons (Schmidt and Catterall, 1986). In *Drosophila*, Para has also been predicted to contain multiple glycosylation sites highly homologous to its mammalian counterpart (unpublished observations). Thus, with its glycosylation nature, Para is well placed as a candidate for Cnx quality control during ER exit.

Several lines of evidences support this hypothesis. First, immunostaining experiments showed that Para co-localizes with Cnx and ER-GFP signals around neuronal nuclei in the adult brains, suggesting that it is potentially under ERAD Cnx quality control. Our Co-IP analysis indicates that Cnx physically interacts with Para. Furthermore, Cnx interacts with synthesized Para protein variants containing distinct glycosylated sites, yet fails to bind to Para protein variant that does not contain any. These results imply that Para is a substrate for Cnx and glycosylation at distinct sites on Para (namely Asn313, 325, 343, 1463, and 1482) are required for Cnx-Para interaction.

Consistent with the hypothesis that Para is a Cnx substrate, our results further suggest that Para protein levels were significantly reduced in the absence of Cnx expression (Figure 2). It is likely that in the absence of Cnx, the ER quality control checkpoint gets loose, allowing the escape of incorrectly folded Para proteins. These malfunctioning Para proteins hinder with proper propagation of action potentials, thus leading to an enhanced defect in locomotor activity in *cnx* and *para* double RNAi animals (Figure 4). To our knowledge, a similar link has been established in mammals, where Cnx interacts with the transmembrane segments of the sodium channel Na_*v*_1.8 and mediates its degradation (Li et al., 2010).

### Cnx regulates para function via calcium buffering

**Figure 7:**
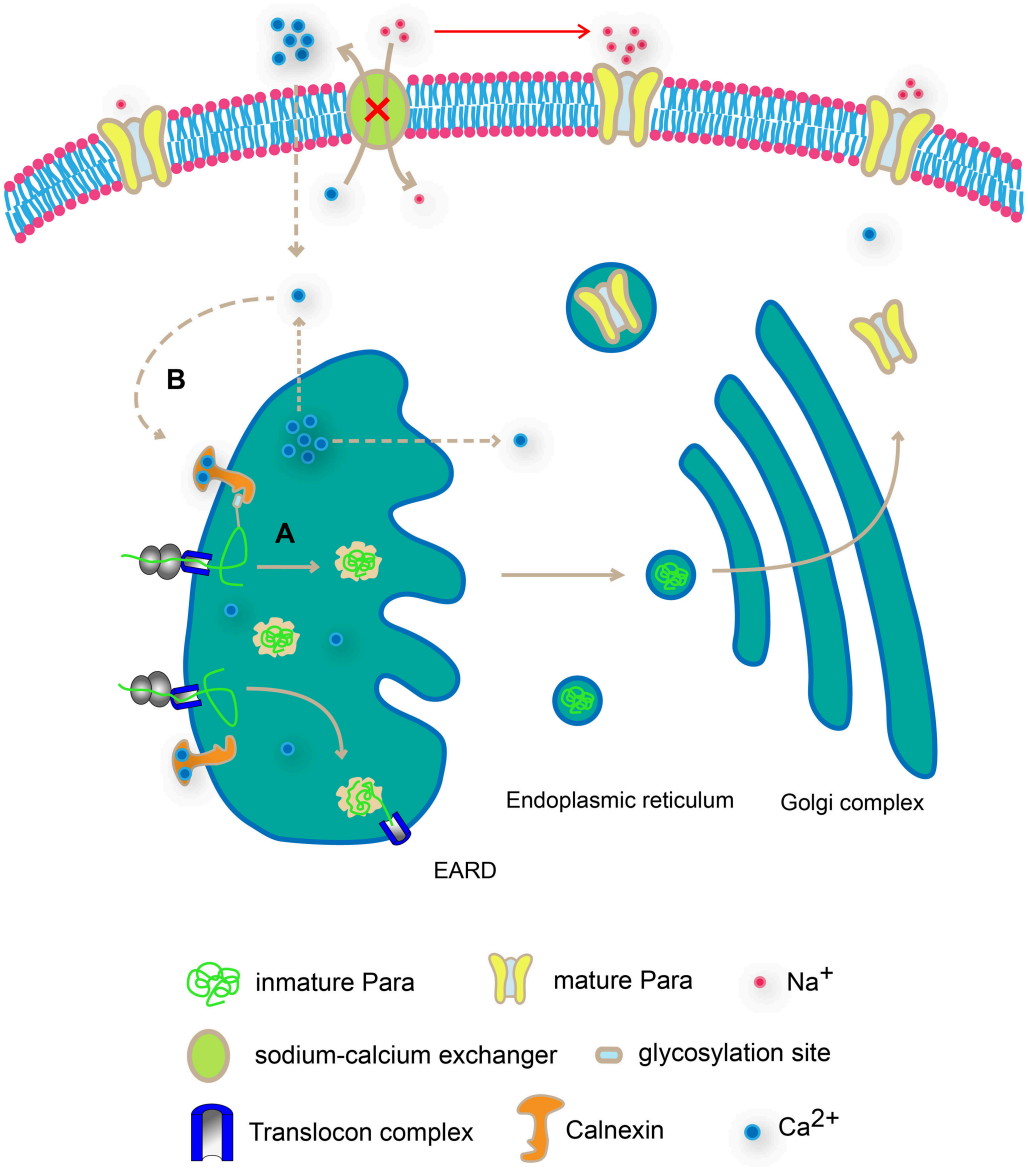
**Schematic diagram on Cnx-mediated Para regulation in two distinct modes**. Cnx regulates Para expression and function via two distinct pathways. First, Cnx interacts with glycosylation sites on Para, suggesting a regular quality control check-up point in ER **(A)**. In addition, Cnx C-terminal domain buffers intracellular Ca^2+^ ion levels, creating a Ca^2+^gradient both inside and outside plasma membrane and the ER membrane **(B)**. This difference in Ca^2+^ ion levels in turn regulates the sodium ion flows via the sodium-calcium exchanger on the plasma membrane or promotes ER vesicular trafficking, both routes affecting Para expression and function.

In addition to the glycosylation-mediated interaction between Cnx and Para, our results support an additional route for Cnx to regulate Para protein levels via its C-terminus. Overexpression of a truncated Cnx protein containing only its C-terminal calcium-binding domain leads to a dramatic upregulation of Para protein levels. As the major lectin domain that mediates Cnx and glycosylated substrate binding resides in the N-terminus, this result was unexpected and leads to the speculation that Cnx regulates intracellular Ca^2+^ ion levels, concomitantly tunes sodium ion flows via a secondary source on the plasma membrane like the sodium-calcium exchanger. By doing so, Cnx modulates Para function via regulating the dynamic of sodium ion flows in the cell. Interestingly, previous report has shown that Cnx acts as a calcium buffering reagent in *Drosophila* eye (Rosenbaum et al., 2006). Thus, in addition to the glycosylation-dependent interaction between Cnx and Para, our analysis on Cnx C-terminus function offers a second possibility on how Cnx controls Para function (Figure 1). Taken together, our results suggest that Cnx regulates Para expression and function via two distinct pathways. First, Cnx interacts with glycosylation sites on Para, suggesting a regular quality control check-up point in ER. Alternatively, Cnx C-terminal domain might regulate Para expression by buffering intracellular Ca^2+^ ion levels (Figure 7)

### Cnx-mediated presynaptic modulation on para function

Our results on Para regulating synaptic growth and function have suggested its critical role during synapse developmental dynamic, involved synaptic circuit, and hence the controlled behavior. As a sodium channel protein, Para controls neuronal excitability, a function reflected by behavioral parameters such as the adult climbing and larval crawling (results in this study and data not shown). Yet, less is known about Para regulation on structural plasticity and synaptic remodeling. Our findings using NMJ pinpoint clearly a role for Para to regulate the differentiation of synaptic boutons, as downregulation of Para expression leads to an increase in the number of undifferentiated boutons containing presynaptic terminals only (ghost boutons). Interestingly, previous studies have revealed that a lack of N-glycosylation leads to NMJ structural overgrowth (Parkinson et al., 2013). This piece of evidence suggests that regulation on Para glycosylation could well be a key factor for its function during synapse formation. Indeed, in our analysis, Cnx plays pivotal roles for the appearance of ghost boutons mediated by Para activity, as exemplified by the decrease in the ghost bouton number upon expression of both *para* and *cnx* RNAi (Figure 5). Similar to the structural evidence, our electrophysiology studies also indicated a Cnx-mediated presynaptic modulation on Para activity as the mEJP frequency and the latency time were both greatly affected in the absence of Cnx and Para expression (Figure 6). These results provide the basis for our behavioral observation and implicate that Cnx and Para regulate locomotor behavior by exerting a function in controlling synaptic differentiation and function.

### Cnx as a player in channelopathies: implication for epilepsy

Channelopathies such as human epilepsy are frequently associated with dysfunction of voltage-gated sodium channels and among the most prevalent diseases in urgent need for therapeutic solution. Modeling epilepsy using *Drosophila* uncovers fundamental biological principles underlying the mechanism of this disease (Parker et al., 2011a; Sun et al., 2012). For instance, a recent study has reported a knock-in mutation of human sodium channel gene *SCN1A* into *Drosophila para*, and by introducing this mutation, temperature-induced seizure phenotype was detected (Sun et al., 2012). This result, together with observations from a number of other studies, has demonstrated that *Drosophila* is an excellent model for studying epilepsy.

Dysregulation of Para function at higher temperature could cause irregular sodium-dependent ion flows and action potential propagation, leading to abnormal firings of neurons as seizures. Our findings on how Cnx misexpression alters the *para*^*ts1*^ recovery profile indicate that Cnx participates in the *para*^*ts1*^-mediated paralysis. These *para*^*ts1*^ results serve as good indicators on how Cnx regulates Para function, a mechanism separable from the glycosylation-dependent regulation as Para protein is mature and its disruption in function due to high temperature is reversible in this scenario. Together with our findings on the Cnx C-terminus plays crucial roles in regulating Para protein levels, we conclude that Cnx, in addition to interact with glycosylated Para, also modulates the dynamic of the calcium and sodium ion flows, which in turn affects Para function.

## Author contributions

XX, CC, TY, JO, YH, LX, and MH conceived and designed the study. XX, CC, TY, JO, and MR performed the experiments. MH wrote the paper. All authors read and approved the manuscript.

### Conflict of interest statement

The authors declare that the research was conducted in the absence of any commercial or financial relationships that could be construed as a potential conflict of interest. The reviewer CB and handling Editor declared their shared affiliation, and the handling Editor states that the process nevertheless met the standards of a fair and objective review.

## Abbreviations

Cnx: Calnexin
Co-IP: co-immunoprecipitation
Elav: Embryonic lethal Abnormal vision
ERAD: endoplasmic reticulum-associated degradation
NMJ: neuromuscular junction
Para: Paralytic
Repo: Reversed polarity
VNC: ventral nerve cord.
