# Supplementary material for: Molecular Chaperone Calnexin Regulates the Function of Drosophila Sodium Channel Paralytic
Source: Front Mol Neurosci. 2017 Mar 7;10:57. doi: 10.3389/fnmol.2017.00057 (PMC5339336; doi:10.3389/fnmol.2017.00057)
Supplement: Supplementary file 1 [file DataSheet1.pdf]

Supplementary Material

Molecular chaperone Calnexin regulates the function of *Drosophila* sodium channel Paralytic

Xi Xiao[1, 2, 3¶], Changyan Chen[4¶], Tian-ming Yu[1, 2, 3], Jiayao Ou[1, 2, 3], Menglong Rui[5], Yuanfen Zhai[1, 2, 3], Yijing He[1, 2, 3], Lei Xue[4], and Margaret S. Ho[1, 2, 3*]

[*]Correspondence:
Email: margaret_ho@tongji.edu.cn (MH)

[¶] X. X and C.C contributed equally to this work

Supplementary Figures

Figure S1

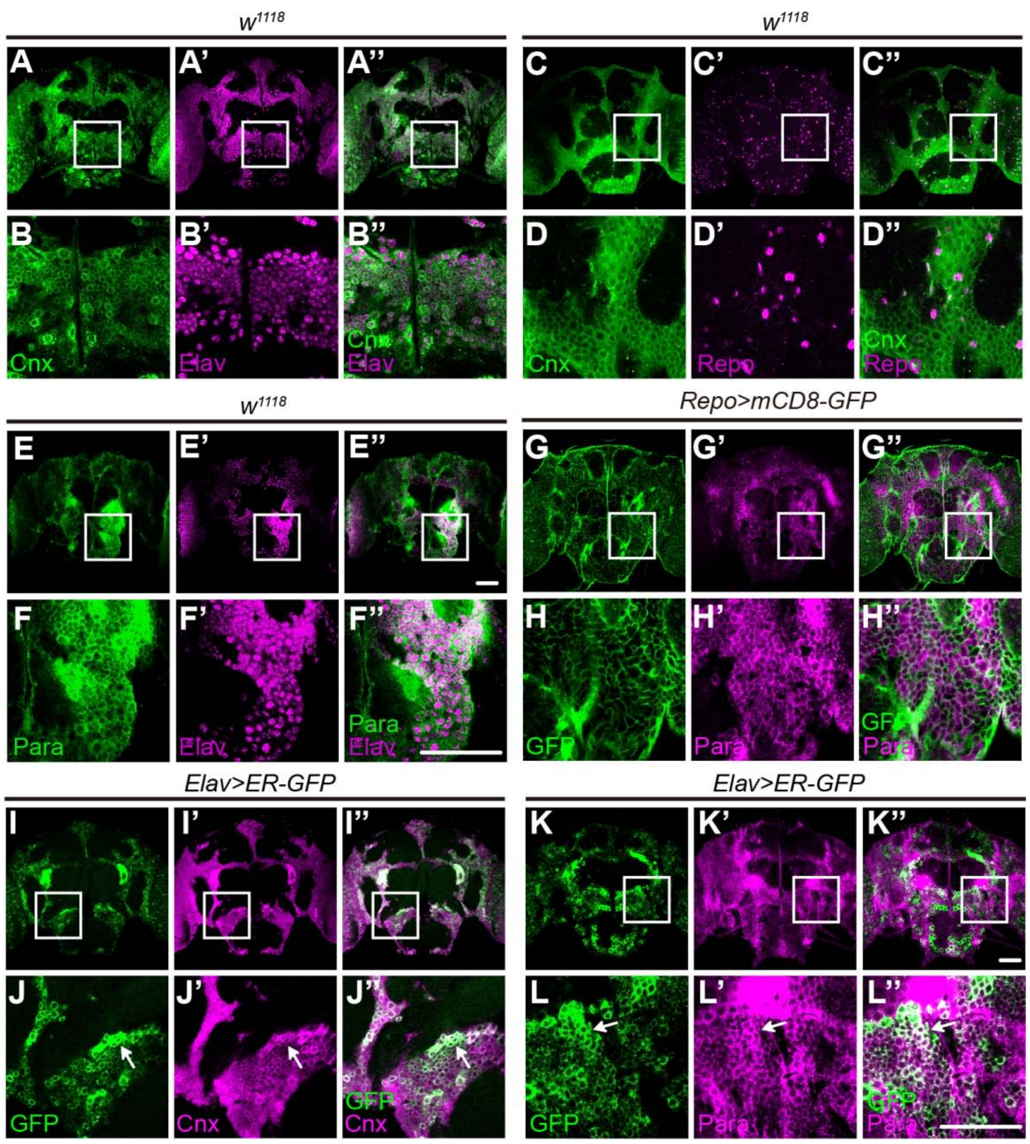

Supplementary Figure 1. Expression analysis of Cnx and Para in *Drosophila* adult brains. (A-D'') Wild-type adult brains were dissected and co-stained with an antibody against the Cnx (green) and Elav (magenta, A-B'') or Repo (magenta, C-D''). Note that Cnx localizes around the Elav-positive nuclei. **(E-F'')** Wild-type adult brains were co-stained with Para (green, E and F) and Elav (magenta, E' and F'). Note a similar around-the-nucleus pattern for Para (F''). **(G-H'')** Adult brains carrying *Repo>mCD8-GFP* were stained with Para (magenta). **(I-L'')** Adult brains expressing *Elav>ER-GFP* were stained with Cnx (magenta, I' and J') or Para (magenta, K' and L'). Note the overlay in signals from both colors (white arrows in J'' and L''). Areas enclosed by the white squares were enlarged and shown directly below. Scale bar: 50 μm.

Figure S2

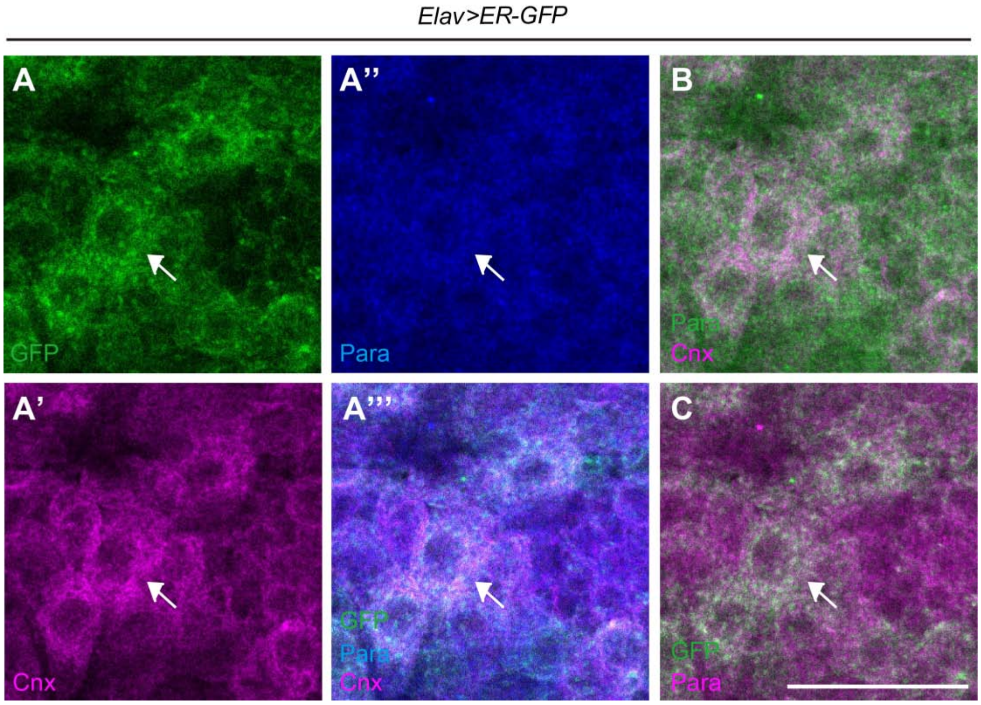

Supplementary Figure 2. Cnx colocalizes and interacts with Para in larval VNCs. The posterior side of larval VNCs carrying *Elav>ER-GFP* were co-stained with Cnx (magenta) and Para (blue in A" and A"', green in B, or magenta in C). Note that some GFP-positive cells were also Cnx- and Para- positive (white arrows in A"' and C) and that Cnx and Para were colocalized (white arrows in A"' and B). Scale bar: 25 μm.

Figure S3

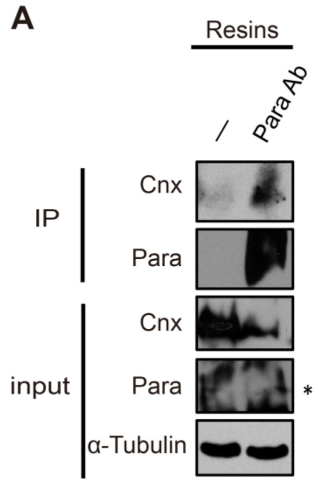

Supplementary Figure 3. Co-IP analysis of Cnx and Para in adult heads. Co-IP analysis was performed using the wild-type adult heads (n>200). Endogenous Cnx proteins were detected in the eluate of Para-conjugated resins. Input control: Cnx, Para, and α-Tubulin. * indicates Para protein.

Figure S4

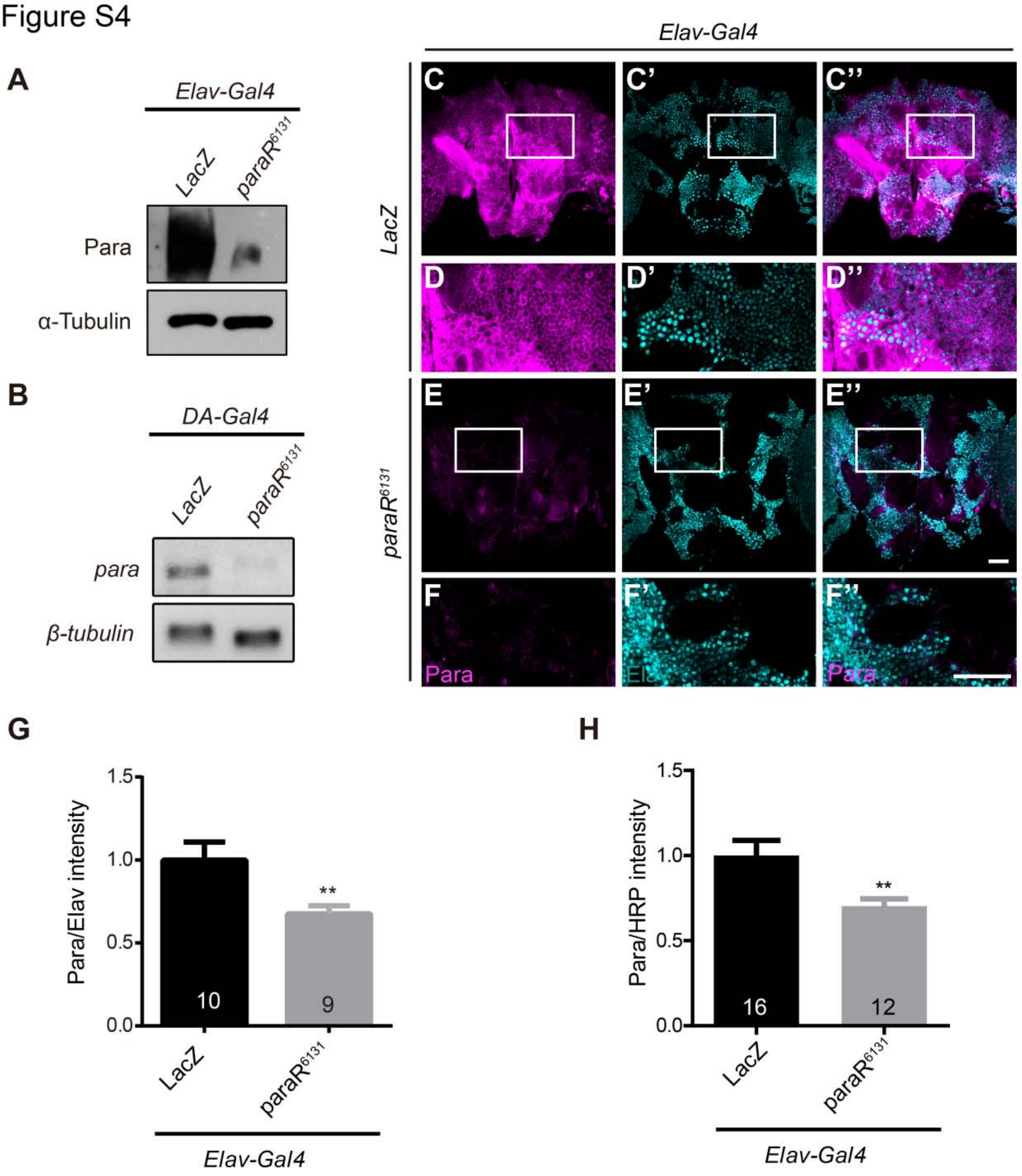

Supplementary Figure 4. Para expression analysis by Western blot, RT-PCR, and immunohistochemistry. (A) Para protein levels were analyzed by the Para antibody generated in this study. Protein extracts were derived from adult heads of *Elav>LacZ* and *Elav>paraR^{6131}* flies (15 heads per lane). Note a decrease in protein levels upon *para* RNAi expression. **(B)** *para* mRNA levels were assessed by RT-PCR when *paraR^{6131}* was expressed. Note a decrease in *para* mRNA upon RNAi expression. **(C-F)** *Elav>LacZ* (C-D") or *Elav>paraR^{6131}* (E-F") adult brains were dissected and stained with Para (magenta) and Elav (cyan). Areas enclosed by the white rectangles were enlarged and shown directly underneath (D-D" and F-F"). Scale bar: 50 μm. **(G)** Para intensities normalized to the internal control Elav in adult brains. The Para/Elav intensities was designated as 1 for *LacZ* control. Note a significant decrease in Para/Elav intensities when *paraR^{6131}* was expressed. **(H)** Para intensities normalized to the internal control HRP in VNC motor axons for *Elav>LacZ* and *Elav>paraR^{6131}*

larvae. The Para/HRP intensities was designated as 1 for *LacZ* control. Note a significant decrease in the intensities when *paraR*^{6131} was expressed. * p<0.05, ** p<0.01, *** p<0.001. Data were shown as mean ± SEM.

Figure S5

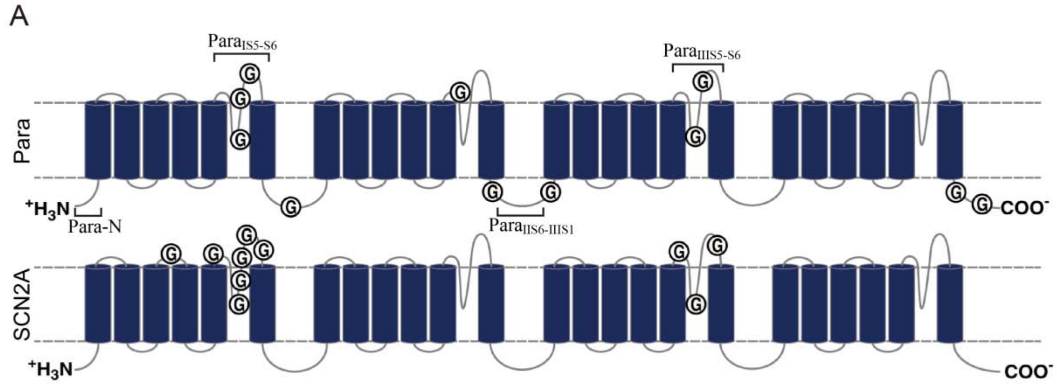

Supplementary Figure 5. A schematic diagram on the potential glycosylation sites of Para and its rat homolog SCN2A. Two Para protein fragments that contain predicted glycosylation sites: Para$_{IS5\text{-}S6}$ from 301aa to 400aa and Para$_{IIIS5\text{-}S6}$ from 1456aa to 1532aa, were designated on the diagram. On the other hand, Para protein fragment Para$_{IIS6\text{-}IIIS1}$ from 1070aa to 1169aa that contains no predicted glycosylation sites was also shown. Para-N contains the first N-terminal intracellular domain was alos shown.

Figure S6

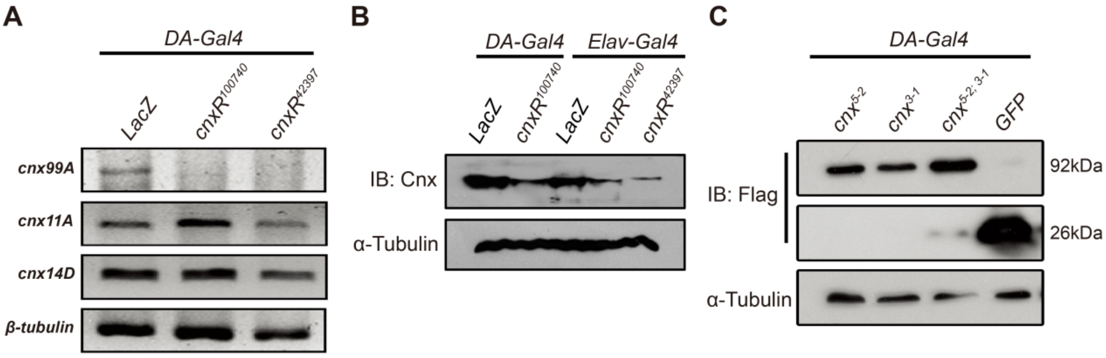

Supplementary Figure 6. Cnx expression analysis by RT-PCR, Western blot, and transgenic overexpression. (A) RNAs extracted from larvae that ubiquitously express *LacZ*, *cnxR*^{100740}, or *cnxR*^{42397} were analyzed by RT-PCR for expression of *cnx* genes: *cnx99A*, *cnx11A*, and *cnx14D*. Control: *β-tubulin*. Note a robust depletion of *cnx99A* mRNA expression. **(B)** Cnx protein levels in adult heads were analyzed by a *Drosophila* Cnx antibody (gift from Nansi Colley). 15 adult heads were used in each lane for the following genotypes: *DA>LacZ*, *DA>cnxR*^{100740}, *Elav>LacZ*, *Elav>cnxR*^{100740}, and *Elav>cnxR*^{42397}. Note a decrease in Cnx protein levels upon RNAi expression. **(C)** Protein extracts from larvae that ubiquitously expressed 3xFlag-Cnx proteins using *DA-Gal4* were homogenized and analyzed by SDS-PAGE. Anti-Flag antibody was used to detect the Cnx and GFP proteins. Control: α-Tubulin.

Figure S7

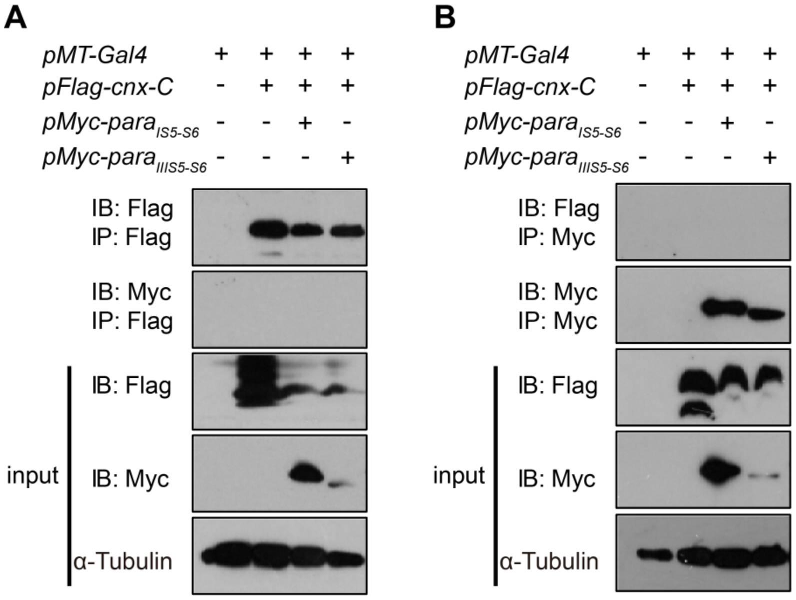

Supplementary Figure 7. C-terminal Cnx fails to interact with Para glycosylation sites. (A and B) Co-IP analysis on the interaction between C-terminal Cnx and two Para protein variants: 6xMyc-Para$_{IS5-S6}$ and 6xMyc-Para$_{IIIS5-S6}$. Note that Cnx-C does not interact with the two Para fragments containing glycosylation sites. Co-IP analyses were done by both Flag- or Myc-antibody conjugated beads.

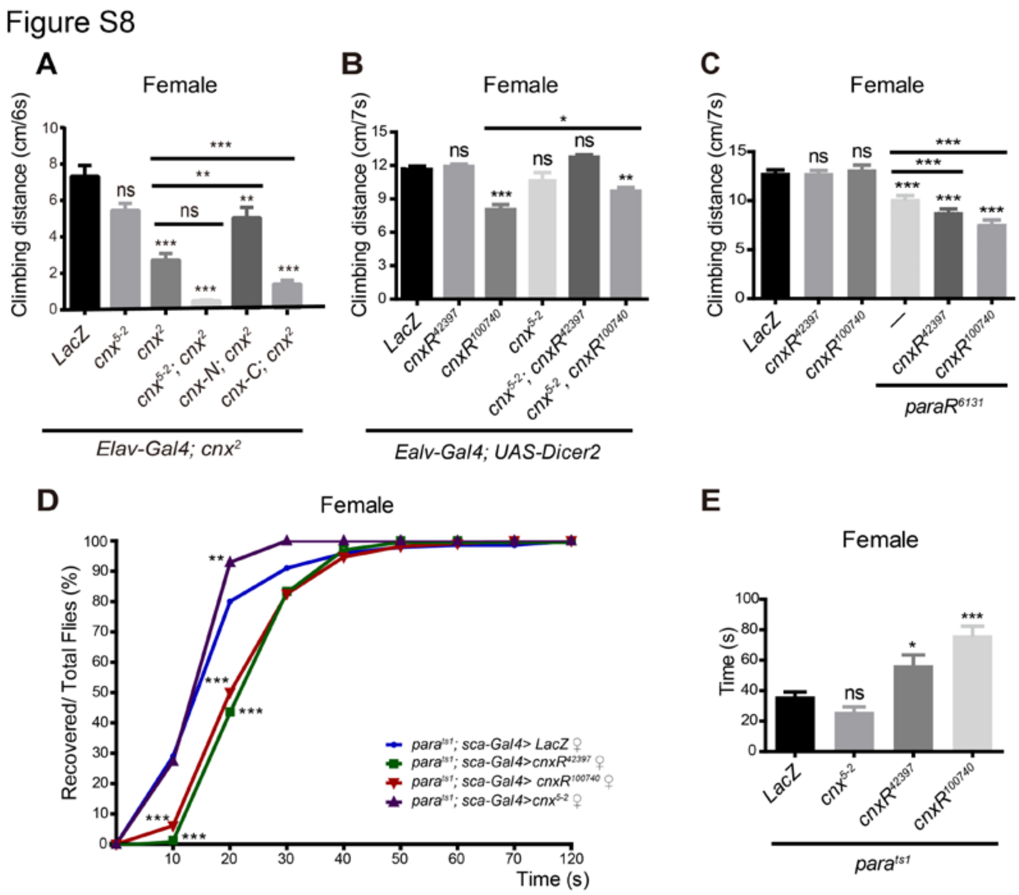

Supplementary Figure 8. Cnx regulates Para-mediated adult climbing and paralysis. Adult climbing ability was analyzed similarly as in Figure 4. Results from female flies were shown in this figure. All genotypes were the same as in Figure 4.

Figure S9

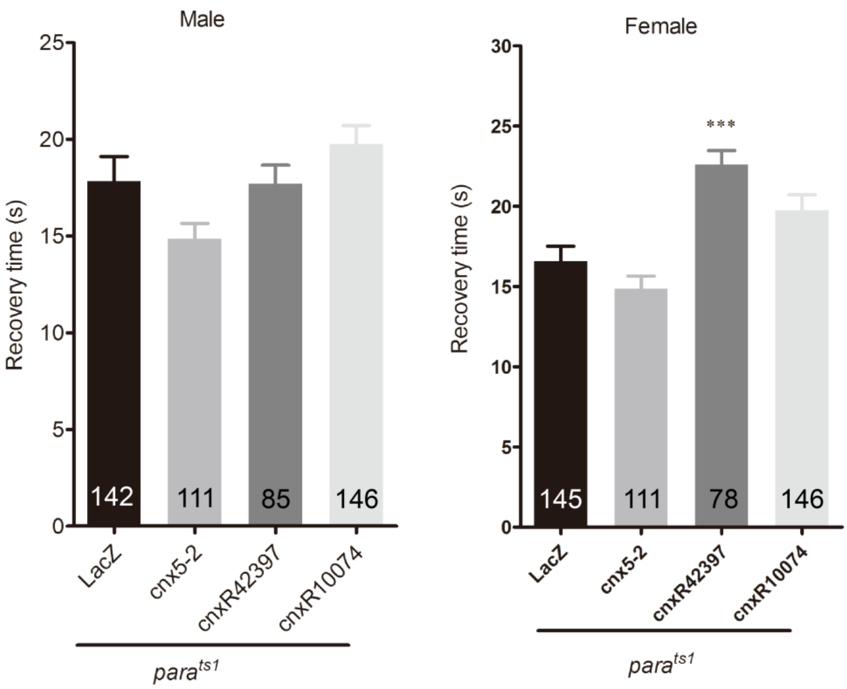

Supplementary Figure 9. Bar graphs with SEM for paralysis experiments. The recovery time was calculated and plotted for the following genotypes: male: *para*^{ts1}; *Sca>LacZ* (n=142), *para*^{ts1}; *Sca>cnx*$^{5\text{-}2}$ (n=111), *para*^{ts1}; *Sca>cnxR*^{42397} (n=85), and *para*^{ts1}; *Sca>cnxR*^{100740} (n=146); female: *para*^{ts1}; *Sca>LacZ* (n=145), *para*^{ts1}; *Sca>cnx*$^{5\text{-}2}$ (n=111), *para*^{ts1}; *Sca>cnxR*^{42397} (n=78), and *para*^{ts1}; *Sca>cnxR*^{100740} (n=146). * p<0.05, ** p<0.01, *** p<0.001, Data were shown in mean±SEM. Total about 100 flies were analyzed for each genotype and three independent experiments were performed.

Supplementary Table 1. Fly stocks used in the current study.

Line	Description
w^{1118}	Wild-type control
Repo-Gal4 (III)	A pan-glial Gal4 driver
Elav-Gal4 (X)	A pan-neuronal Gal4 driver
UAS-mCD8-GFP (II)	UAS control
UAS-LacZ (II)	UAS control
UAS-GFP.KDEL (III)	GFP with a C terminal ER retention signal
UAS-Dicer2 (II)	Enhance RNAi effectiveness
UAS-cnxR^{42397} (III)	*cnx* RNAi
UAS-cnxR^{100740} (II)	*cnx* RNAi
UAS-3xFlag-cnx$^{5-2}$ (II)	Transgenic flies overexpressing Cnx
UAS-3xFlag-cnx$^{3-1}$ (III)	Transgenic flies overexpressing Cnx
cnx^{2} (III)	cnx mutant
UAS-3xFlag-cnx-N (II)	Transgenic flies overexpressing N-terminal Cnx (1-489aa)
UAS-3xFlag-cnx-C (II)	Transgenic flies overexpressing C-terminal Cnx (509-605aa)
para^{ts1} (X)	*para* temperature sensitive allele
UAS-paraR^{6131} (III)	*para* RNAi

Supplementary Video 1. Cnx regulates the recovery kinetic of *para*^{ts1}-mediated paralysis. A video was recorded to detect the *para*^{ts1} flies recovering from paralysis at the non-permissive temperature. Note that *para*^{ts1} flies recovered in a shorter time in the presence of Cnx overexpression, whereas *para*^{ts1} flies in the absence of Cnx expression rarely move or climb during the same time frame.

Supplementary Materials and Methods

Detailed protocols on adult brain dissection

At least 10 adult flies of 3 days old for each genotype were collected in a 1.5 ml Eppendorf tube placed on ice. The collected flies were temporarily disabled by the low temperature and transferred to the dissection plate filled with droplets of 1X PBS solution. Use the dissection needles (Minucie/500 pieces No. 15, Czech republic) to hold the fly in a ventral side up position to remove the trophi carefully, put the flies in an Eppendorf tube with fixation buffer (4% formaldehyde in 1X PBS), and rotate the tube on the nutator for 40 mins. Next, use pipets to remove the 4% formaldehyde fixation buffer and wash the flies with 1 ml 1X PBT (0.3% Triton in 1X PBS) for 15mins, repeated three times at room temperature. Flies were then transferred to the dissection plate for removing the brains from the head cuticle by forceps. Removed and fixed brains were collected in a new Eppendorf tube with 1X PBT.

Detailed protocols on adult brain immunostaining

Fixed brains were incubated in solution containing the primary antibody in 1X PBT and 5% NDS, then rotated on the nutator at 4 °C overnight. Optimal antibody titer used vary among different antibodies and a solution cocktail containing the antibody in 1X PBT and 5% NDS was prepared for equal distribution into tubes with different sample sets. This preparation helps to minimize the difference that could occur during the staining process. After overnight, the primary antibody solution was removed and sample brains were washed with 1ml 1X PBT for 10 mins, repeated three times at room temperature. Next, sample brains were incubated with solution containing the secondary antibody in 1X PBT and 5% NDS, rotated on the nutator for 2 hours in room temperature, then washed with 1X PBT for 10 mins, repeated three times. Stained brains were then mounted on slides and ready for microscopic observations.

Detailed protocols on confocal image acquisition

Standard user protocols for Leica LSP5 confocal microscope were followed throughout the image acquisition. For adult brains, we used a 40X oil objective to take a Z-stack projection of 15-20 sections in step size 1μm. The beginning and the end of the Z-stack sections were chosen based on Para staining fluorescence. Moreover, all images were taken in the same settings including contrast, gains, zoom, pinhole, etc.

Detailed protocols on quantification of fluorescent intensities

Leica Microsystems software (LAS Af lite) was used to quantify the intensities of fluorescent colors acquired by different channel wavelengths. Upon opening the software, the quantification module was chosen to designate 3 regions of interest (ROIs) on each side of the brain (total 6 ROIs). The module measured the fluorescent intensities from each ROI in arbitrary units and divided the number over total area to give a mean value for each ROI. Total 6 mean values were obtained for Para staining intensities and each was divided by the mean value obtained for the staining of an internal control (i.e., HRP) in the same ROI to give the mean value of Para/HRP. At least 10 brains were analyzed for each genotype. There were at least 60 mean values of Para/HRP obtained for each experimental group. These values were normalized to the averaged mean value obtained for the control group (i.e., *Elav>LacZ*, averaged mean value of Para/HRP). The control group was always designated as 1 after normalization. Data were shown ±SEM and P-values were calculated using unpaired T-test between two groups and one-way ANOVA with Bonferroni multiple comparison test among three groups or above. ns means no significance. * $p<0.05$, ** $p<0.01$, *** $p<0.001$.
